# The role of biogeographical barriers on the historical dynamics of passerine birds with a circum‐Amazonian distribution

**DOI:** 10.1002/ece3.10860

**Published:** 2024-03-06

**Authors:** Sergio D. Bolívar‐Leguizamón, Fernanda Bocalini, Luís F. Silveira, Gustavo A. Bravo

**Affiliations:** ^1^ Seção de Aves Museu de Zoologia da Universidade de São Paulo São Paulo Brazil; ^2^ Laboratório de Zoologia de Vertebrados, Departamento de Ciências Biológicas, Escola Superior de Agricultura “Luiz de Queiroz” –ESALQ– Universidade de São Paulo Piracicaba Brazil; ^3^ Sección de Ornitología, Colecciones Biológicas, Instituto de Investigación de Recursos Biológicos Alexander von Humboldt Claustro de San Agustín Villa de Leyva, Boyacá Colombia; ^4^ Museum of Comparative Zoology and Department of Organismic and Evolutionary Biology Harvard University Cambridge Massachusetts USA

**Keywords:** climatic fluctuations, demographic modeling, environmental gradients, Forest Refugia hypothesis, Thamnophilidae, ultraconserved elements

## Abstract

Common distributional patterns have provided the foundations of our knowledge of Neotropical biogeography. A distinctive pattern is the “circum‐Amazonian distribution”, which surrounds Amazonia across the forested lowlands south and east of the basin, the Andean foothills, the Venezuelan Coastal Range, and the Tepuis. The underlying evolutionary and biogeographical mechanisms responsible for this widespread pattern of avian distribution have yet to be elucidated. Here, we test the effects of biogeographical barriers in four species in the passerine family Thamnophilidae by performing comparative demographic analyses of genome‐scale data. Specifically, we used flanking regions of ultraconserved regions to estimate population historical parameters and genealogical trees and tested demographic models reflecting contrasting biogeographical scenarios explaining the circum‐Amazonian distribution. We found that taxa with circum‐Amazonian distribution have at least two main phylogeographical clusters: (1) Andes, often extending into Central America and the Tepuis; and (2) the remaining of their distribution. These clusters are connected through corridors along the Chaco–Cerrado and southeastern Amazonia, allowing gene flow between Andean and eastern South American populations. Demographic histories are consistent with Pleistocene climatic fluctuations having a strong influence on the diversification history of circum‐Amazonian taxa, Refugia played a crucial role, enabling both phenotypic and genetic differentiation, yet maintaining substantial interconnectedness to keep considerable levels of gene flow during different dry/cool and warm/humid periods. Additionally, steep environmental gradients appear to play a critical role in maintaining both genetic and phenotypic structure.

## Graphical abstract

This study found similarities at populational, phylogenetic, and evolutionary levels among four taxonomic groups of passerine birds with a circum‐Amazonian distribution. The presence of Refugia result of climatic oscillations during the Pleistocene was the main drive in the diversification of these circum‐Amazonian taxa.

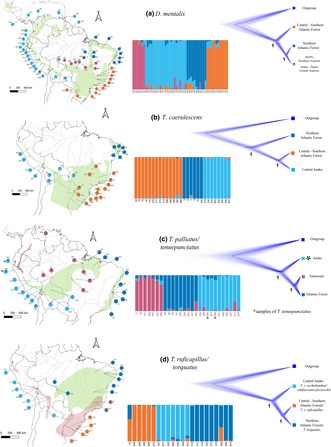

## 1 INTRODUCTION

The South American avifauna is widely known as the richest and most diverse in the world (Stotz et al., 1996), and extensive research has been devoted to explaining the mechanisms underlying its outstanding diversity (Burney & Brumfield, 2009; Haffer, 1969; Harvey et al., 2020; Ribas et al., 2012; Sick, 1967; Silva et al., 2019; Smith et al., 2014). Comparative analyses of genetic variation in co‐distributed taxa have featured prominently among those studies, and they have generated important insights into the diversification and biogeographical history of the South American biota (Bocalini et al., 2021; Carnaval et al., 2009; Harvey et al., 2017; Johnson et al., 2023; Lima‐Rezende et al., 2022; Musher et al., 2022; Naka & Brumfield, 2018; Silva et al., 2019; Thom et al., 2021; Thom, Xue, et al., 2020). However, whereas ample research has focused on the mechanisms generating and maintaining diversity in species‐rich areas, such as the Amazonian and the Andean realms (Carvalho et al., 2021; Gergonne et al., 2022; Hazzi et al., 2018; Miranda et al., 2021, among others), the mechanisms responsible for the diversity of regions with less homogeneous habitats remain poorly understood.

Some South American bird species and species complexes exhibit rather odd geographical distributions that are neither correlated with environmental boundaries nor affected by geographical barriers that are known to separate many other species. One such pattern is the so‐called “circum‐Amazonian distribution”, in which forest bird species or species complexes occur right around the Amazon rainforest (Figure 2). This pattern was originally noted in the geographical range of a few birds, namely *Platyrinchus mystaceus*, *Dysithamnus mentalis*, *Phyllomyias burmeisteri*, *Elaenia albiceps*, *E*. *parvirostris*, and the *E*. *obscura*/*sordida* complex (Remsen et al., 1991). Subsequent work identified this pattern in other bird species, including *Asemospiza* grassquits (Bates, 1997), *Myiothlypis* warblers (Lovette, 2004), *Synallaxis* spinetails (Batalha‐Filho, Irestedt, et al., 2013), and *Pionus* parrots (Ribas et al., 2007). Additionally, another bird species such as *Stilpnia cayana*, *Thamnophilus caerulescens*, and a few *Cercomacra* antbirds partially fit the pattern, given that they only occupy portions of this distribution (Bolívar‐Leguizamón et al., 2020; Savit & Bates, 2015; Tello et al., 2014). A circum‐Amazonian distribution has also been identified in other organisms such as insects (Canals & Johnson, 2000; Erwin, 2000; Irmler, 2009) and plants (Knapp, 2002; Prado & Gibbs, 1993). Patterns of parallel distribution among independent lineages usually imply concurrent underlying phenomena that have shaped their distribution. These encompass a wide range, from geological events such as the formation of rivers or the uplift of mountainous chains to ecological interactions among groups. These interactions may include habitat competition between circum‐Amazonian species and their Amazonian peers. Additionally, other factors as climatic oscillations might have shaped the distributions of these organisms promoting isolation and secondary contact to fit within this observed pattern.

The increasing number of molecular markers in the genomics era has allowed the estimation of demographic parameters and the statistical comparison of biogeographical scenarios for taxa with “unusual” distributional patterns (Bocalini et al., 2023; Bolívar‐Leguizamón et al., 2020; Corbett et al., 2020; Thomé et al., 2021). Savit and Bates (2015) used molecular and niche modeling analyses for *Stilpnia cayana* (Thraupidae) and suggested a southern origin for the taxon in the Brazilian Cerrado with subsequent expansion through the Andes (Bolivia region) into the Tepuis and northeastern Brazil (via the “dry forest arc”). However, it is unlikely that this scenario explains the current distribution of other circum‐Amazonian taxa, due not only to the idiosyncrasies of each lineage (habitat specificity, response to environmental changes) but also since the geological and ecological complexity of the distribution cannot be summarized using the history of only one taxon. Classical hypotheses of diversification can be evoked to try to explain the distribution of circum‐Amazonian organisms, such as the Forest Refugia (Haffer, 1969; Vanzolini & Williams, 1970) and the Riverine Barrier hypotheses (Sick, 1967; Wallace, 1854); two of the most tested hypotheses about lineage diversification in Neotropics (da Rocha & Kaefer, 2019; Garzón‐Orduña et al., 2015; Mascarenhas et al., 2019; Moncrieff et al., 2023). In the same way, the “Gradient” hypotheses could explain this pattern (see Endler, 1982; Ortiz et al., 2018; Wang & Bradburd, 2014). Formal analyses testing these hypotheses have not been implemented for circum‐Amazonian organisms. Here, we use a comparative phylogenomic approach to characterize patterns of genetic population structure, gene flow, and demographic history of four antbird species and species complexes exhibiting a circum‐Amazonian distributional pattern and try to fit these data with the premises of these diversification hypotheses.

The species in this study included (a) the Plain Antvireo (*Dysithamnus mentalis*), a taxon with a complete circum‐Amazonian distribution pattern, occurring in a wide range from southeastern Mexico to south Brazil right around Amazonia, in the understory and mid‐story levels of humid, lower, and montane evergreen forest, including populations inhabiting moist “terra firme” and várzea forest (Figure S1a) (Zimmer & Isler, 2020). (b) The Variable Antshrike (*Thamnophilus caerulescens*), with an incomplete circum‐Amazonian distribution, inhabiting the tropical evergreen forest edge from Peru south along the Andes to Argentina and in Eastern Brazil. Its habitat includes areas of evergreen forests, second‐growth woodland, and patches of thickets and trees in open regions (Figure S1b) (Zimmer & Isler, 2003). (c) The Chestnut‐backed and the Lined Antshrikes (*Thamnophilus palliatus* and *Thamnophilus tenuepunctatus*, respectively) are known to form a complex with shallow genetic divergence (Harvey et al., 2020) despite their obvious differences in male plumage. Their joint distribution represents a complete circum‐Amazonian complex, covering the montane forested areas of northern Andes (*T. tenuepunctatus*) and the forested regions of the Central Andes, southern Amazonia, and the Atlantic Forest (*T. palliatus*, Figure S1c) (Zimmer et al., 2020; Zimmer & Isler, 2019). Finally, (d) the Rufous‐capped and the Rufous‐winged Antshrikes (*Thamnophilus ruficapillus* and *Thamnophilus torquatus*) also form a complex (Harvey et al., 2020) that shows an atypical circum‐Amazonian distribution. *Thamnophilus ruficapillus* is formed by populations in forested montane areas of the Central (subspecies *jaczewskii* and *marcapatae*) and Southern Andes (subspecies *subfasciatus* and *cochabambae*), whereas the Atlantic population (nominate subspecies) inhabits shrubby vegetation from Bahia (Brazil) to Argentina. *Thamnophilus torquatus* occurs in the Cerrado and riparian thickets from Eastern Brazil to Northeastern Bolivia and Paraguay (Figure S1d) (del Hoyo et al., 2020; Zimmer & Isler, 2017).

Specifically, we used genome‐wide single nucleotide polymorphism (SNPs) from flaking regions of ultraconserved elements (UCEs) to estimate parameters of genetic population structure and demographic history for each species, and we assessed contrasting biogeographical scenarios explaining the circum‐Amazonian distribution based on the presence of geographical barriers and environmental gradients. We tested scenarios with parameters reflecting the presence of Quaternary refugia (Refugia Hypothesis, Haffer, 1969 and Vanzolini & Williams, 1970), including divergences during the Quaternary, population expansion and bottlenecks with variation in population size, and migration—secondary contact—among clusters. Alternative scenarios included no gene flow among clusters after differentiation, constant population size, and divergence times congruent with the formation of geographical barriers.

As shown in the paragraph above, and despite being considered circum‐Amazonian, the four taxa analyzed here do not share the same specific habitats. Due to this, it is expected that the best scenarios for each taxon show some differences in some of their estimated parameters. For example, under a Refugia scenario (sensu stricto), the best models for species with the distributions in humid forested areas (e.g., *D. mentalis*) would be marked by “strong” events of populational expansion/bottleneck and secondary migration, while for species inhabiting evergreen forests (e.g., *T. caerulescens*) the chosen models would not show these parameters in that extend. In the same way, groups with mountainous distributions (the Andean and Atlantic Forest populations) would not be fitted in models where Refugia is the main force of diversification, and possibly, geographical barriers could be more effective.

## 2 MATERIALS AND METHODS

### 2.1 Sampling and DNA extraction

We gathered 288 vouchered tissue samples from ornithological collections (see Acknowledgments), covering the majority of the species ranges (Figure S1; Table S2). Previously published data and specimens sequenced were included specifically for this study (Bolívar‐Leguizamón et al., 2020; Harvey et al., 2020). As outgroups, we used sequences of *Dysithamnus leucostictus* (for *D*. *mentalis*), *Thamnophilus aethiops* (for *T*. *caerulescens*), *Thamnophilus zarumae* and *T. multistriatus* (for *T*. *palliatus*/*tenuepunctatus*) and *Thamnophilus doliatus* (for *T*. *ruficapillus*/*torquatus*) (see Table S1, Harvey et al., 2020). For newly sequenced samples, we extracted total genomic DNA from muscle samples using the PureLink® Genomic DNA Mini kit (Invitrogen Inc.) following the manufacturer's guidelines, and we quantified genomic DNA concentrations using a Qubit 2.0 fluorometer with the dsDNA BR assay kit (Life Technologies, Inc).

### 2.2 Mitochondrial DNA

As a preliminary assessment of population structure, divergence times, and demography for each study group, we sequenced the mitochondrial gene *NADH dehydrogenase 2* (ND2) for all samples and at least one outgroup (see Table S1). We amplified and sequenced the ND2 gene using standard PCR and Sanger sequencing protocols as described in Brumfield and Edwards (2007). We edited sequences and checked for stop codons or anomalous residues using Geneious v. 9.1. (Kearse et al., 2012). We aligned sequences with the MAFFT v.7 multiple alignment plugin (Katoh & Standley, 2013) as implemented in Geneious. Final alignments contained 1041 bp (See Table S1 for details). Newly generated ND2 sequences were deposited in GenBank (accession numbers PP105012—PP105070 and PP119346—PP119416 for *D. mentalis*; PP111986—PP112008 for *T. palliatus*; PP105071 for *T. tenuepunctatus*; PP106098—PP106122 for *T. ruficapillus*/*torquatus*). ND2 sequences for *T. caerulescens* (MT079216—MT079269) were gathered from Bolívar‐Leguizamón et al. (2020).

We built median‐joining haplotype networks (Bandelt et al., 1999) as implemented in POPART 1.7.2. (Leigh & Bryant, 2015) to examine the relationships among mitochondrial haplotypes for each group. Outgroups and short ingroup sequences were excluded and matrices were trimmed to exclude positions containing missing data (see Table S1). Also, we estimated genetic diversity (π, θ_W_) and Tajima's D to summarize molecular variation and to infer populational changes using the untrimmed alignments. We performed an Analysis of Molecular Variance (AMOVA) to detect population differentiation using the packages *pegas* (Paradis, 2010), *adegenet* (Jombart, 2008), and *poppr* (Kamvar et al., 2014) in R 3.6 (R Core Team, 2021).

We selected the best substitution model for each species (see Table S3) using the corrected Akaike Information Criterion (AICc; Hurvich & Tsai, 1989) as implemented in jModeltest2 v2.1.6. (Darriba et al., 2012) on the Cipres Science Gateway V 3.3 (Miller et al., 2010). To inform the test of historical scenarios (section 2.8), we estimated a time‐calibrated gene tree within a Bayesian framework implemented in the program BEAST2 v2.4.4 (Bouckaert et al., 2014). Following Nabholz et al. (2016), we used a body mass correction to estimate mean substitution rates for each species (Table S3). We used a strict molecular clock and a Coalescent Constant Population prior with no restrictions on tree shape and a randomly generated tree as a starting tree. We ran analyses for a total of 50 million generations with a sampling frequency of 1000. We determined that replicate analyses converged when effective sample size values were greater than 400 using Tracer 1.7.1 (Rambaut et al., 2018). Using TreeAnnotator v2.4.4 (Bouckaert et al., 2014; Drummond et al., 2012) and a burn‐in of 30%, we generated maximum clade credibility (MCC) with a posterior probability limit of 50%.

### 2.3 Ultraconserved elements sequence capture

Based on the mtDNA results and seeking to further explore divergence and demographic history, we sequenced UCEs for a subset of our samples (Faircloth et al., 2012; McCormack et al., 2012) (Table S2). We sent at least 1 μg of genomic DNA of each sample to RAPiD Genomics (Gainesville, FL) to build and enrich genomic libraries and conduct Illumina sequencing. Libraries were enriched from 2386 UCE loci that targeted a set of 2560 probes (Faircloth et al., 2012, Tetrapods‐UCE‐2.5 K version 1; Microarray, Ann Arbor, MI), following an open‐source protocol (available at www.ultraconserved.org). Samples were multiplexed at 192 samples per lane on a 125 bp paired‐end Illumina HiSeq 2500 run (sample pooling was performed at Rapid Genomics with samples from other projects), yielding an average coverage of 18.6x per sample.

We followed the *Phyluce* pipeline v1.6 (Faircloth, 2016, https://github.com/faircloth‐lab/phyluce) to process the raw reads and assemble contigs corresponding to target loci. Initially, Illumiprocessor 2.0.7 (Faircloth, 2013) and Trimmomatic 0.32 (Bolger et al., 2014) were implemented to trim adapters, barcodes, and low‐quality regions. We used Trinity 2.0.6 (Grabherr et al., 2011) to perform the assembly (script *phyluce_assembly_assemblo_trinity*). To avoid including markers of different ploidy, we identified, extracted, and removed Z‐linked UCEs from the assemblies using Blast 2.7.7 (Altschul et al., 1990; Camacho et al., 2009). We removed Z‐linked loci from downstream analyses to sample autosomal coalescent histories, thereby avoiding biases in haplotype calling resulting from differential population sex ratios. Finally, we implemented the script *phyluce_assembly_match_contigs_to_probes* to match the assembled contigs to the UCE probes (uce‐2.5 k‐probes.fasta). Table S4 summarizes the number of trimmed reads and assembled contigs. UCE data is available on NCBI Genbank (BioProject: PRJNA1064987).

### 2.4 SNP calling

For each taxon, we extracted SNPs from the UCE alignments using the methods described by Bolívar‐Leguizamón et al. (2020) and Harvey et al. (2016), which are largely based on Phyluce (Faircloth, 2016). We extracted SNPs using the script *phyluce_assembly_match_counts*. The number of loci in the matrices can be accessed in Table S5. Because the amount of missing data was not substantial (max. NA value was 10.41%), we did not filter the matrices. Thus, we created a *fasta file with the loci extracted from the incomplete matrix of each species (*phyluce_assembly_explode_get_fastas_file*), and we chose the reference sequence for each study group based on coverage and mean length of recovered contigs (script *phyluce_assembly_get_trinity_coverage*, and *phyluce_assembly_get_fasta_lengths*). We used bwa 0.7.7 (Li & Durbin, 2009) to map raw reads from the samples for each species to their respective reference (Li, 2013), and we used SAMtools 0.1.19 (Li et al., 2009) and Picard (Broad Institute, 2019, http://broadinstitute.github.io/picard/) to create *bam files and identify duplicates from the PCRF. We used the GATK 3.8.0 (McKenna et al., 2010) to extract indels, SNPs, and phase SNP alleles. To avoid using linked SNPs, we retained only one random SNP per locus to generate both the complete and incomplete matrices (script *rand_var_per_chr.pl*, https://github.com/caballero/Scripts). We exported the resulting *vcf files into other formats for the downstream demographic analyses (*nexus, *sfs.gz).

### 2.5 Population structure

We inferred population clustering in our SNP datasets using two complementary approaches: (1) principal component analyses (PCA) as implemented in the R packages *adegenet* and *ape* (Jombart, 2008; Popescu et al., 2012); and (2) sparse non‐negative matrix factorization (sNMF) using the R package *LEA* (Frichot et al., 2014; Frichot & François, 2015). We used the PCA approach to visualize the variation in our data, and a discriminant analysis of principal components (DAPC; Jombart et al., 2010) to estimate the number of genetic clusters in the data using the R package *adegenet*. We implemented the sNMF using six α regularization parameter values (1, 10, 50, 100, 500, 1000), *K* values of 1–10, 100 runs per *K* value, and the minimum cross‐entropy as TRUE to estimate the best number of *K*. To compare with the mtDNA data, we estimated genetic diversity (π, θ_W_) and Tajima's D for the SNPs matrices using the R package *sambaR* (de Jong et al., 2021).

### 2.6 Species trees

To infer the phylogenetic relationships among clusters recovered in population structure analyses, we used a species delimitation approach as implemented in SNAPP (Bryant et al., 2012). We ran three million iterations in two runs (see the “SNAPP_scenarios.docx” in the Supplementary Material), using default values for the backward (*u*) and forward (*v*) mutation rates and the value λ with a gamma distribution (α = 2). We checked that the effective sample size (ESS) values >400 using Tracer 1.7.1 (Rambaut et al., 2018). Using BEAST2 (Bouckaert et al., 2014), we inferred and visualized the posterior distribution of species trees. Specifically, we merged *trees files using *logcombiner*, and visualized them *densitree* (Bouckaert & Heled, 2014).

### 2.7 Spatial distribution of the genetic diversity

To identify putative geographical barriers, we used estimated effective migration surfaces *EEMS* (Petkova et al., 2016). EEMS identifies patterns of genetic diversity that deviate from a null expectation of isolation by distance (Wright, 1943) by examining matrices of geographical and genetic distances and dividing the landscape into demes (https://github.com/dipetkov/eems). We implemented three runs of 20 million iterations for each species with groups of 300, 500, and 700 demes (for *D. mentalis* we ran 40 million iterations). We then plotted the results using the R package *reemsplots2* (https://github.com/dipetkov/reemsplots2).

### 2.8 Demographic history and shared divergences

We used the software *momi2* (Kamm et al., 2019, https://github.com/popgenmethods/momi2) to infer possible demographic scenarios for each species. *Momi2* (Moran Models for Inference) infers demographic histories by fitting the observed value of the site‐frequency spectrum (SFS) data to its expected value in a composite likelihood framework (a coalescent framework). Here, we used the SNPs matrices to extract the SFS file (*momi.extract_sfs*) and used the *momi.DemographicModel* function to build the models, and tested the model fit with the function *model.optimize*, which yields a log‐likelihood value. In this way, multiple demographic models were assessed and the best‐fit scenario was selected using the Akaike Information Criterion (AIC, Akaike, 1973). For all models, we used a mutation rate of 2.5 × 10^−9^ substitutions per site per generation (Nadachowska‐Brzyska et al., 2015) and a generation time of 2.33 years, following estimates for other thamnophilids (Maldonado‐Coelho, 2012; Thom et al., 2018). We implemented 100 runs to avoid suboptimal results. Based on the run with the highest likelihood for each model, we evaluated the relative weight of the best model using the Akaike information criterion (AIC). We rank the tested models by estimating the ΔAIC scores and Akaike weights (ωi, Burnham & Anderson, 2004). We ran 100 bootstrap simulations to estimate confidence intervals (CI) of the parameters in the most informative model. Finally, based on the most informative models for each species, we looked for common scenarios across taxa potentially underlying the circum‐Amazonian distribution.

**FIGURE 1:**
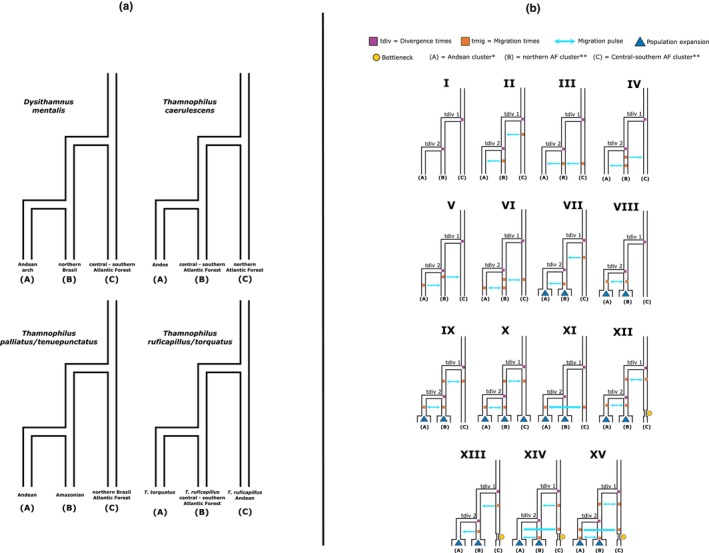
(a) Schematic of the groups used in the demographic models tested in momi2 for the genomic clusters identified for the four species via PCA and DAPC, all models are constructed based on a three‐population scenario. (b) Complete list of models tested for the species analyzed (from I to XV). This list covers events of migration and divergence events only, and more complex models with more events included (models with migration, divergence times, bottlenecks, etc.). Model parameters are noted as follows: tdiv: divergence times between populations; tmig: migration times between clusters; blue arrows: Migration pulse; yellow circle: bottleneck event; blue triangle: expansion event; A: Andean cluster; B: northern Atlantic Forest cluster; and (c) central‐southern Atlantic Forest cluster. *For the *T. ruficapillus*/*torquatus* model the (a) terminal represents the *T. torquatus* cluster. **For these models, terminals (b) and (c) represent the clusters identified in (a).

We tested multiple models with different demographic scenarios for the genetic clusters identified in the population structure analyses (see Figure 1a). We tested between 12 and 15 models (I–XV, Figure 1b) for each species using different ranges for the estimated parameters. For each species, initial models were simplest (fewer parameters, for instance, the estimation of the divergence times), increasing in complexity as more parameters to estimate migration events, bottlenecks, and populational expansions were included in subsequent models. Parameters such as individual migration waves, bottlenecks, and population expansions were estimated in each model, and each model had distinct migration times and directions (Figure 1b). Figure 1b depicts parameters used in the models, with models I–VI showing individual migration events and divergence times, whereas the following models show multiple migration pulses, and events bottlenecks and expansion (models VII–XV). We tested Pleistocene Refugia models that included (a) divergences during the Quaternary, (b) events of populational expansion and bottlenecks that could produce considerable levels of variation in population size in the clusters due to the expansion/retractions of forested circum‐Amazonian regions, and (c) events of migration—secondary contact—as a consequence of the expansions/retractions of these forests that could allow the isolation and reconnection of the circum‐Amazonian populations after their divergences. Alternatively, the “geographical barriers” scenarios included (a) no gene flow among clusters after differentiation, (b) constant populational size, and (c) divergence times congruent with the rise of the proposed geographical barrier (as a primary barrier). For the circum‐Amazonian distribution, barriers such as the São Francisco and the Paraná‐Paraguay basins, and the Andes mountainous chain could be proposed as effective barriers for the taxa analyzed here. A “null” scenario included constant populational size, no migration, and non‐Quaternary divergence times (“a free divergence time” estimation without constraints).

We ran the software *ecoevolity* (Oaks, 2019) to identify shared events of divergence across study groups. This software allows testing multiple models while modifying the number of divergence events and mutation rates. Model assumptions include constant population size along each branch, no migration, and similar relative mutation rates among species. Our goal was to compare whether geographically congruent breaks (Andes–Atlantic Forest; northern Atlantic Forest–central/southern Atlantic Forest) among the four taxa were best explained by a single divergence event or by multiple divergence events over time. We implemented two types of analysis: (1) for the Andes–Atlantic Forest divergence (all four taxa), and (2) for the northern Atlantic Forest–central/southern Atlantic Forest divergence (except for *T. palliatus*/*tenuepunctatus*). We ran 50,000 iterations for each comparison, testing multiple models based on the concentration parameter of the “event model prior” proposed in the *ecoevolity* tutorial (http://phyletica.org/ecoevolity/tutorials/gecko‐divergences.html).

## 3 RESULTS

### 3.1 Analyses of mtDNA

The mtDNA alignments contain 967 bp for *D. mentalis*, 825 bp for *T. caerulescens*, 1025 bp for *T. palliatus*/*tenuepunctatus*, and 1025 bp for *T. ruficapillus*/*torquatus*. We found considerable haplotype diversity within each taxon (Figure S2). Tajima's *D* values suggest recent populational expansions for all taxa except the *T. ruficapillus*/*torquatus* complex (see Tables S6 and S7 mtDNA and UCE statistics, respectively). We recovered both *D. mentalis* and *T. caerulescens* as reciprocally monophyletic in mtDNA gene trees (Figure S3), whereas the *T. palliatus*/*tenuepunctatus* and the *T. ruficapillus*/*torquatus* complexes, as currently defined, were recovered as non‐monophyletic (Figure S4). We found that *T. tenuepunctatus* is embedded within the Andean clade of *T. palliatus*. Also, we found that *T. ruficapillus* from the southern Atlantic Forest is more closely related to *T. torquatus* than to populations of *T. ruficapillus* in the Andes (Figure S4a, b). Stem ages of all groups were in the Late Pliocene (Piacenzian, Figures S3 and S4), and divergences within groups of each species were in the Middle Pleistocene (0.7–0.1 ma), except for the *T. ruficapillus*/*torquatus* complex, which was in the Calabrian (1.8–0.7 ma).

### 3.2 Population structure in circum‐Amazonian antbirds for UCE data

**FIGURE 2:**
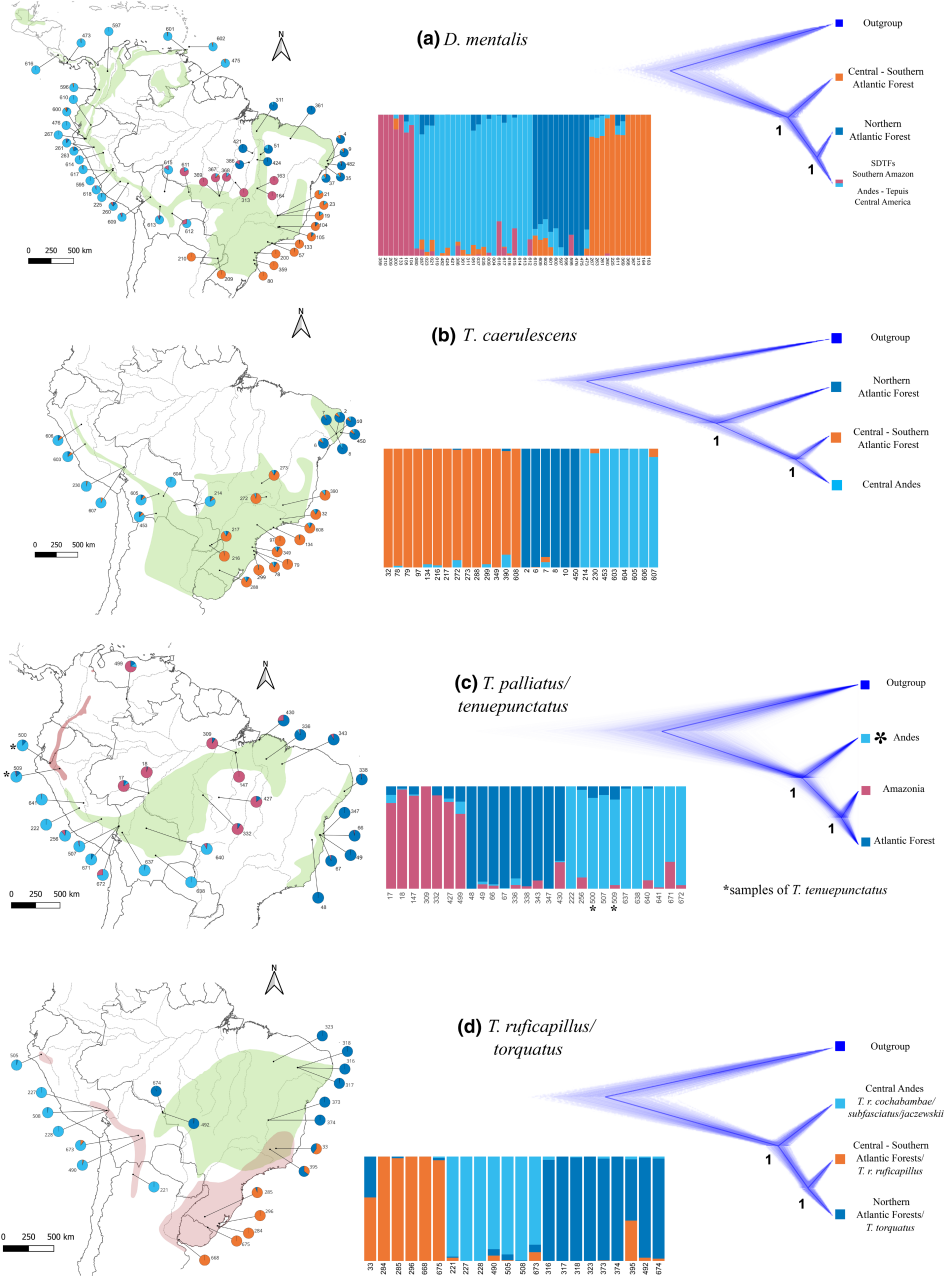
Population structure and species tree analyses of four circum‐Amazonian species. (a) *D. mentalis* (1848 SNPs). (b) *T. caerulescens* (2036 SNPs). (c) *T. palliatus*/*tenuepunctatus* complex (1855 SNPs). (d) the *T. ruficapillus*/*torquatus* complex (1845 SNPs). Left: Maps with pie charts representing admixture coefficients (K with lowest cross‐entropy values) for each sample as quantified by sNMF. Right: Cladograms of species tree inferences (SNAPP analyses). Numbers at nodes represent posterior probability values of the 50% Maximum Clade Credibility Tree. Colored squares represent populations as colored on maps.

For the UCE data, the number of loci recovered per matrix was: *Dysithamnus mentalis* (1848 loci, 9% of missing data—NA) *Thamnophilus caerulescens* (2036 loci, 1.5% NA), *T. palliatus*/*tenuepunctatus* complex (1855 loci, 10.41% NA), and *T. ruficapillus*/*torquatus* complex (1845 loci, 7.5% NA). Based on the geographical distributions and population structure of the four taxa, we identified at least two common population clusters in these circum‐Amazonian antbirds: (1) an Andean group, often extending into Central America and the Tepuis; and (2) the remaining of their distribution (Figure 2; Figure S5). Nonetheless, we uncovered idiosyncratic genomic structure and admixture patterns within these clusters across taxa. The first two principal components in the DAPC analysis together explained 80% of the between‐group variance (Figure S5). Both DAPC and sNMF analyses indicated a genetic cluster restricted to central and southern Atlantic Forest for each *D. mentalis*, *T. caerulescens*, and the *T. ruficapillus*/*torquatus* complex (Figure 2a, b, d). We found admixture between this cluster and the northern Atlantic Forest cluster in the *T. ruficapillus*/*torquatus* complex and with the central Andean regions for *T. caerulescens* (Figure S5).

A northern Atlantic Forest cluster was identified only in *T. caerulescens* corresponding to samples from the Atlantic Forest of the Brazilian states of Alagoas and Pernambuco (“Pernambuco Center of Endemism”, Figure 2b). In *D. mentalis* and the *T. palliatus*/*tenuepunctatus* complex, samples from this region and those from eastern Amazonia grouped together (Figure 2a–c). We found evidence of admixture between these latter clusters and the central‐southern Atlantic Forest cluster in *T. caerulescens*, and the adjacent western‐southern Amazonian clusters in *D. mentalis* and the *T. palliatus*/*tenuepunctatus* complex.

We identified Andean clusters in all taxa, but these were not as geographically confined as in the previous two clusters. For example, in *D. mentalis* we identified an Andean cluster that encompasses populations from the northern/central Andes, the Tepuis, and Central America (Figure 2a). Populations of *T. caerulescens* and the *T. ruficapillus*/*torquatus* complex shared a central Andean cluster composed of individuals from northern Argentina and Bolivia to central Peru (Figure 2b–d). The *T. palliatus*/*tenuepunctatus* complex (Figure 2c) also had an Andean cluster, but limited sampling hampered defining its geographical limits.

sNMF analyses revealed substantial levels of admixture among clusters, primarily in individuals from southeastern Bolivia and southern Peru. We found a second area of admixture in individuals from the northern and central Andes. These individuals showed considerable admixture with individuals from forested areas in Brazil (*D. mentalis*) or with individuals from central Andes, Central America, or the Tepuis. Individuals in the *T. palliatus*/*tenuepunctatus* complex showed considerable admixture among the three inferred clusters. In *D. mentalis* and the *T. palliatus*/*tenuepunctatus* complex, sNMF identified very close values of cross‐entropy for different numbers of clusters (Figure S6; Tables S8–S11).

Here, we summarize our structure results to specify the cluster configurations used in the posterior analyses. DAPC and sNMF show three clusters for *T. caerulescens*; four for *D. mentalis*; three for the *T. palliatus*/*tenuepunctatus* complex (DAPC = 2; sNMF = 3); and three for *T. ruficapillus*/*torquatus* complex (DAPC = 4; sNMF = 3). Here, the level of incongruence among the number of clusters identified between DAPC and sNMF is probably due to that the sNMF algorithm takes into account the possibility of gene flow after divergence and the presence of individuals with admixed genotypes, while the DAPC analysis does not. DAPC might potentially recognize clusters comprising only admixed individuals or merge clusters that exhibit significant levels of admixture.

For the SNAPP analysis (Section 3.3 in Results), we used a “three‐cluster” configuration for all groups based mainly on the idea that the individuals with high levels of admixture have to be identified as hybrids and not as a separated group from their “parent” clusters. Additionally, we ran scenarios using the “discordant” results from DAPC to test these alternative assemblages (Figure S7). Specifically for *D. mentalis*, we used a four‐cluster setting congruent with the PCA/sNMF results (Figure S7a), however, the most supported scenario was a topology with three clusters (as presented in Figure 2a).

For the *momi2* analysis (Section 3.5 in Results), we used a “three‐cluster” configuration for all groups based on the most supported scenario from SNAPP. In the case of the *D. mentalis* database (*k* = 4), we run *momi2* using this configuration because one of the groups does not have the ideal number of individuals to estimate the populational statistics (from the SFS data) and run successfully *momi2* (the Seasonally Dry Tropical Forests “SDTFs”—Southern Amazon group in Figure 2). In *momi2* is necessary to delete “hybrid” individuals from all populations, which reduces the number of individuals per population. Additionally, the most supported topology from SNAPP is a tree with three populations; central‐southern Atlantic Forest; northern Atlantic Forest; and Andean + STDFs populations (see PP values—*Posterior Probability*—in Figure S7).

### 3.3 Species trees

The species tree of *D. mentalis* inferred with SNAPP placed *mentalis* of the southern Atlantic Forest (subspecies *mentalis*) as sister to a clade comprising all other taxa in the complex. Within the latter clade, a group containing all populations in the Andes and Central America was recovered as sister to *affinis* from Central Brazil, and this clade was in turn recovered as sister to *emiliae* from the northeastern Atlantic Forest and eastern Amazonia (Figure 2a). In *T. caerulescens*, the Andean and the southern Atlantic Forest genetic clusters formed a clade sister to the northern Atlantic Forest cluster (subspecies *cearensis* in Bolívar‐Leguizamón et al., 2020; Figure 2b). In the *T. palliatus*/*tenuepunctatus* complex, the genetic cluster formed by Amazonian *palliatus* populations east of the Tocantins River and the Atlantic Forest (subspecies *vestitus*) is sister to all other Amazonian *palliatus* populations (nominate and *puncticeps*) including birds on the Orinoco River in Colombia. In turn, this clade is sister to a cluster formed by Andean populations, including both *tenuepunctatus* and subspecies *similis* and *puncticeps* (Figure 2c). Surprisingly, we found that *T. ruficapillus* as currently defined is not monophyletic. Andean populations of the *T. ruficapillus* (subspecies *cochabambae*, *subfasciatus*, and *jaczewskii*) were recovered as sisters to a clade comprising the Atlantic Forest population of *T. ruficapillus* (nominate) and *T. torquatus* (Figure 2d). All species tree topologies had strong statistical support (PP > 0.99). Figure S7 shows alternative topologies tested for all taxa, some of them not consistent with our main results.

### 3.4 Shared events of divergence in circum‐Amazonian passerines

**FIGURE 3:**
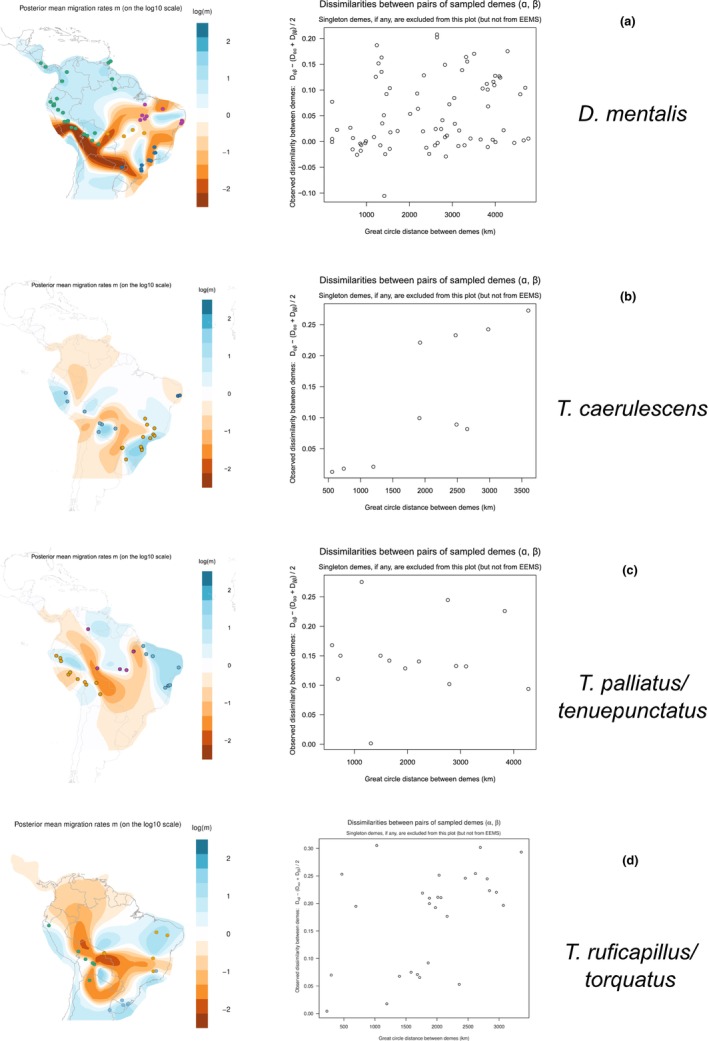
Estimated Effective Migration Surface (EEMS) of four circum‐Amazonian antbird species. (a) *D. mentalis*. (b) *T. caerulescens*. (c) the *T. ruficapillus*/*torquatus* complex. (d) the *T. ruficapillus*/*torquatus* complex. Left: posterior mean of effective migration surface, the color bar representing the effective migration rate on a log10 scale relative to the average over its entire range. Blue colors represent areas of high migration or dispersal corridors, whereas orange regions represent regions with low migration or dispersal barriers. Right: dissimilarities between pairs of sampled demes.

The results of the EEMS were largely congruent with the putative geographical barriers we identified in the population structure analyses (DAPC and sNMF). The barrier placed between the northern and the central/southern regions of the Atlantic Forest was identified as having a lower‐than‐expected migration area for *T. caerulescens* and *D. mentalis* (Figure 3a, b). Similarly, a barrier between the Andes and the Atlantic Forest was recovered in the *T. caerulescens*, *D. mentalis*, and the *T. ruficapillus*/*torquatus* complex, including the northern areas of the Chacoan ecoregions plus the Chiquitano Dry Forest and the Pantanal (Figure 3a, b, d). For the *T. palliatus*/*tenuepunctatus* complex, two zones with lower‐than‐expected migration rates fit the geographical clusters identified with sNMF and DAPC, one separating the Atlantic forests + extreme east Amazonian population from the Amazonian cluster and a second one splitting the latter from the Andean populations (Figure 3c).

### 3.5 Demographic history and shared evolutionary events

**FIGURE 4:**
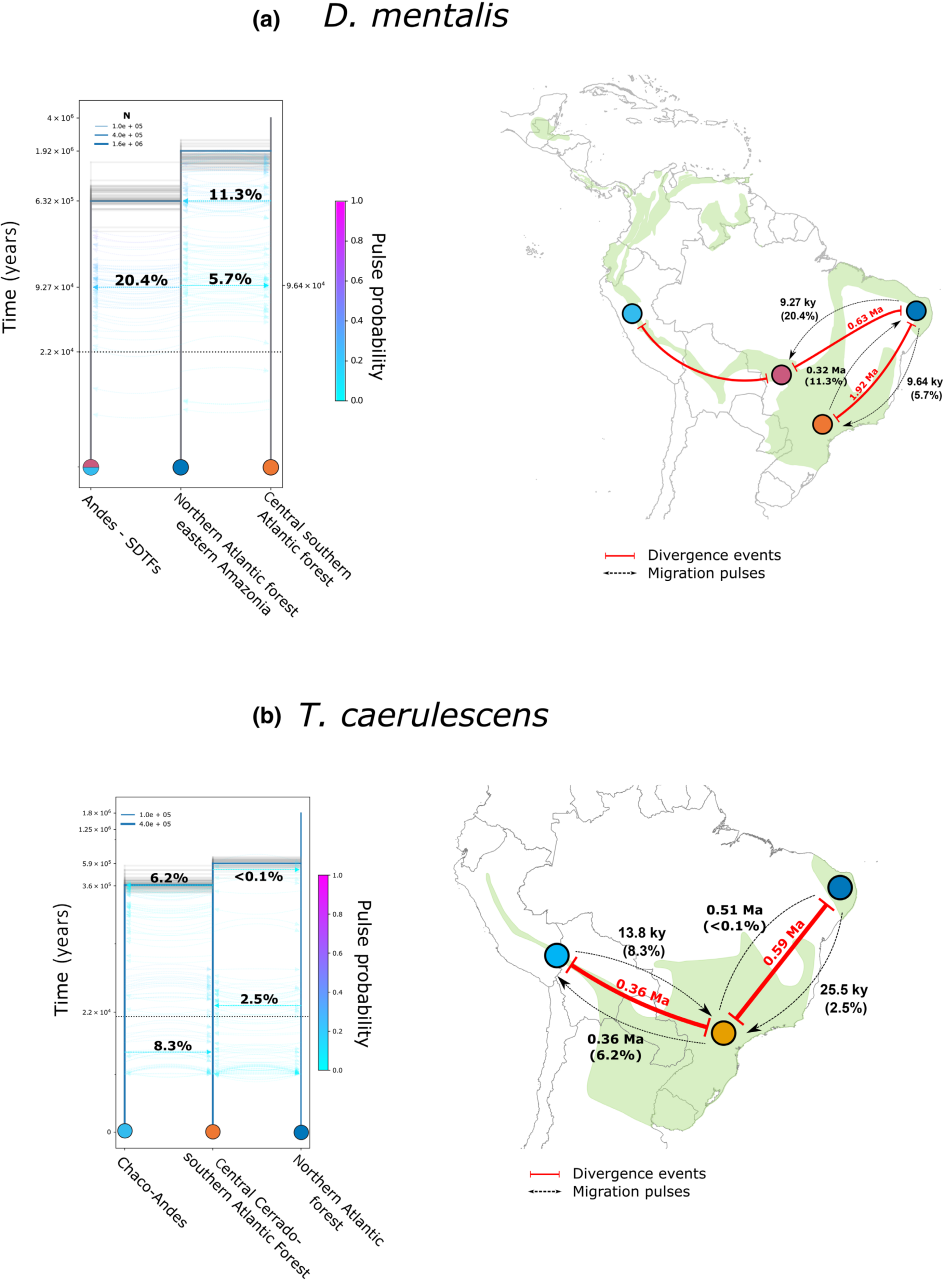
Maps showing the putative divergence and migration times between the main areas of this study as inferred by momi2 for (a) *D. mentalis* and (b) *T. caerulescens* (modified from Bolívar‐Leguizamón et al., 2020). In the maps, divergence times are represented by solid red lines. Migration times are represented by dashed black lines. In the drawing models, migration pulses are dashed blue arrows. Values represent the chronological order estimated across the two species. Values associated with migration events (light blue arrows) represent the percentage of Ne that migrated (For interpretation of the references to color in this figure legend, the reader is referred to the web version of this article).

**FIGURE 5:**
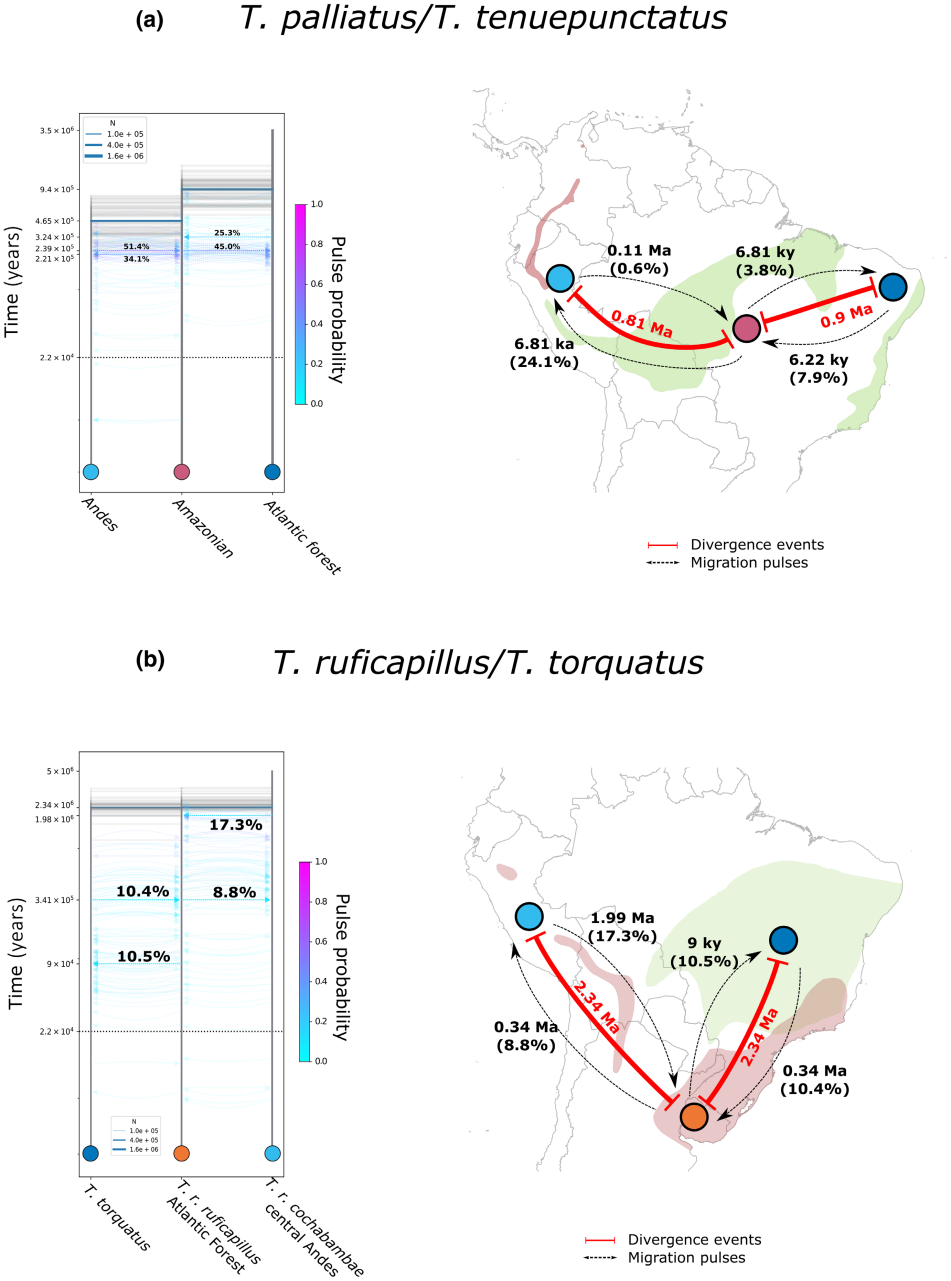
Maps showing the putative divergence and migration times between the main areas of this study as inferred by momi2 for (a) *T. palliatus*/*tenuepunctatus* complex; (b) *T. ruficapillus*/*torquatus* complexes. In the maps, divergence times are represented by solid red lines. Migration times are represented by dashed black lines. In the drawing models, migration pulses are dashed blue arrows. Values represent the chronological order estimated across the two species. Values associated with migration events (light blue arrows) represent the percentage of Ne that migrated (For interpretation of the references to color in this figure legend, the reader is referred to the web version of this article).

We identified common patterns among the most informative models for each species (Figures 4 and 5; Tables 1, 2, 3, 4). In general, demographic models supported bidirectional migration between populations with relatively low migration rates (maximum values 51%–25% in the *T. palliatus*/*tenuepunctatus* complex) and without expansion or bottleneck events. For the *T. palliatus*/*tenuepunctatus* complex and *D. mentalis*, migration rates were higher from Amazonia to the Andes than in the opposite direction. In *T. caerulescens* and the *T. ruficapillus*/*torquatus* complex, highest migration rates occurred between the southern Atlantic Forest and the Andes, where individuals close to the contact zone showed high levels of admixture (see Figure 2). Initial divergence times for all taxa fell within the Pleistocene, ranging from early Gelasian to Middle Pleistocene (*T. ruficapillus*/*torquatus* complex = 2.34 Ma; *D. mentalis* = 1.92 Ma; *T. palliatus*/*tenuepunctatus* complex = 0.94 Ma; *T. caerulescens* = 0.74 Ma), similar to the divergence time estimates inferred based on mtDNA (Figures S3 and S4). Confidence intervals for these estimates are shown in Table 5. Pulses of migration for all taxa except *T. caerulescens* happened prior to the Last Glacial Maximum (Figures 4 and 5).

**TABLE 1:** Information theory statistics and ranking of the 13 demographic models evaluated with momi2 using the matrix (1848 SNPs) of *D. mentalis*.

Rank	Type model	Model number	Log‐lik	n_parameters	ΔAIC	ω_i_
1	Divergences, migrations, and expansions	X^a^	−5262.392335^a^	14^a^	0^a^	1^a^
2	Divergences and migrations	III	−5261.258098	16	1.731526292	0.420730351
2	Divergences and migrations	IV	−5480.279081	18	443.7734919	4.32E‐97
3	Divergences, migrations, and expansions	IX	−5262.817181	16	4.84969321	0.088491692
4	Divergences and migrations	II	−5275.603138	12	22.42160665	1.35E‐05
5	Divergences, migrations, expansions, and bottleneck	XII	−5279.918669	16	39.05266805	3.31E‐09
6	Divergences	I	−5309.276383	8	81.76809762	1.76E‐18
7	Divergences and migrations	VI	−5486.576572	18	456.3684745	7.96E‐100
8	Divergences and migrations	V	−5407.541805	18	298.2989407	1.68E‐65
9	Divergences, migrations, and expansions	VII	−5491.428656	22	474.0726424	1.14E‐103
10	Divergences, migrations, and expansions	VIII	−7152.90404	22	3797.023411	0
12	Divergences, migrations, and expansions	XI	−18294.94633	22	26081.10799	0
13	Divergences, migrations, expansions, and bottleneck	XIII	−9709.965268	22	8911.145868	0

*Note*: The most informative models included parameters related to divergence and migration events only. See the number of models in Figure 2b.

^a^ Chosen model.

**TABLE 2:** Information theory statistics and ranking of the 16 demographic models evaluated with *momi2* using the matrix (2036 SNPs) of *T. caerulescens*.

Rank	Type model	Model number	Log‐lik	n_parameters	ΔAIC	ω_i_
1	Migrations and divergence	VI^a^	−7290.17^a^	13^a^	0^a^	1^a^
2	Migrations and divergence	V	−7299.86	9	42.70805956	5.32E‐10
3	Migrations and divergence	III	−7323.52	9	90.02627891	2.83E‐20
4	Migrations and divergence	IV	−7345.25	9	133.4742895	1.04E‐29
5	Migrations and divergence	I	−7383.25	4	201.4848944	1.77E‐44
6	Migrations and divergence	II	−7386.64	5	208.2665891	5.96E‐46
7	Migrations, divergence, and expansion	IX	−7388.48	16	233.9450474	1.58E‐51
8	Migrations, divergence, expansion, and bottlenecks	XII	−7394.48	16	245.9444395	3.93E‐54
9	Migrations, divergence, and expansion	X	−7394.5	16	245.9804667	3.85E‐54
10	Migrations, divergence, and expansion	XI	−7522.21	11	491.4091924	1.96E‐107
11	Migrations, divergence, and expansion	VIII	−7528.52	11	504.026249	3.57E‐110
12	Migrations, divergence, and expansion	VII	−7574.6	11	596.1774969	3.48E‐130
13	Migrations, divergence, expansion, and bottlenecks	XIV	−7621.89	13	706.7514342	3.40E‐154
14	Migrations, divergence, expansion, and bottlenecks	XV	−7724.95	19	896.8894352	1.75E‐195
15	Migrations, divergence, expansion, and bottlenecks	XIII	−7807.85	11	1062.687182	1.74E‐231

*Note*: The most informative models included parameters related to divergence and migration events only. See the number of models in Figure 2b.

^a^ Chosen model.

**TABLE 3:** Information theory statistics and ranking of the 16 demographic models evaluated with *momi2* using the matrix (1855 SNPs) of *T. palliatus*/*tenuepunctatus* complex.

Rank	Type model	Model number	Log‐lik	n_parameters	ΔAIC	ω_i_
1	Migrations and divergence	VI^a^	−5579.311612^a^	13^a^	0^a^	1^a^
2	Migrations and divergence	IV	−5580.702926	18	4.782627777	0.091509372
3	Migrations and divergence	V	−5580.702925	18	6.782625722	0.033664451
4	Migrations and divergence	II	−5580.702926	18	6.782628723	0.033664401
5	Migrations, divergence, and expansion	VII	−5580.702926	18	8.782628494	0.012384442
6	Migrations, divergence, and expansion	VIII	−5580.702932	18	8.782641215	0.012384364
7	Migrations, divergence, and expansion	IX	−5583.571452	15	10.51968011	0.005196136
8	Migrations, divergence, and expansion	XI	−5589.231892	18	21.84056017	1.81E‐05
9	Migrations, divergence, expansion, and bottlenecks	XIV	−5596.241702	20	31.86018025	1.21E‐07
10	Migrations, divergence, expansion, and bottlenecks	XII	−5596.133101	0	35.64297771	1.82E‐08
11	Migrations, divergence, and expansion	X	−5598.420216	17	40.21720792	1.85E‐09
12	Migrations and divergence	III	−5600.743277	17	48.86333114	2.45E‐11
13	Migrations, divergence, expansion, and bottlenecks	XIII	−5605.351173	20	50.07912211	1.33E‐11
14	Migrations and divergence	I	−5629.561523	5	84.49982329	4.48E‐19
15	Migrations, divergence, expansion, and bottlenecks	XV	−6177.53322	28	1210.443216	1.43E‐263

*Note*: The most informative models included parameters related to divergence and migration events only. See the number of models in Figure 2b.

^a^ Chosen model.

**TABLE 4:** Information theory statistics and ranking of the 12 demographic models evaluated with *momi2* using the matrix (1845 SNPs) of *T. ruficapillus*/*torquatus* complex.

Rank	Type model	Model number	Log‐lik	n_parameters	ΔAIC	ω_i_
1	Migrations and divergence	VI^a^	−6273.820533^a^	13^a^	0^a^	1^a^
2	Migrations, divergence, and expansion	VII	−6274.55217	15	5.463274405	0.0651126
3	Migrations and divergence	III	−6274.999944	16	8.358821986	0.015307521
4	Migrations and divergence	V	−6277.374519	15	11.10797085	0.003871995
5	Migrations and divergence	II	−6282.69762	15	21.75417302	1.89E‐05
6	Migrations and divergence	IV	−6288.002384	14	30.3637023	2.55E‐07
7	Migrations, divergence, and expansion	XI	−6286.667405	16	31.69374272	1.31E‐07
8	Migrations, divergence, and expansion	VIII	−6307.841439	12	66.04181228	4.56E‐15
9	Migrations, divergence, expansion, and bottlenecks	XII	−6340.19633	16	138.7515946	7.42E‐31
10	Migrations, divergence, and expansion	IX	−6354.465715	12	159.290364	2.57E‐35
11	Migrations, divergence, and expansion	X	−6354.835164	12	160.0292625	1.78E‐35
12	Migrations and divergence	I	−6374.94489	5	186.248714	3.60E‐41

*Note*: The most informative models included parameters related to divergence and migration events only. See the number of models in Figure 2b.

^a^ Chosen model.

**TABLE 5:** Demographic parameters estimate inferred with momi2 for the best‐ranked models of each taxon and 95% confidence intervals.

Parameters	*D. mentalis*	*T. caerulescens*	*T. palliatus*/*T. tenuepunctatus*	*T. ruficapillus*/*T. torquatus*
tdiv (A)/(B)	0.632 Ma (0.321–1.495)	0.366 Ma (0.31–0.563)	0.465 Ma (0.242–0.814)	2.342 Ma (1.071–2.846)
tmig (A) → (B)	‐	0.014 Ma (0.010–0.396)	0.239 Ma (0.035–0.511)	0.090 Ma (0.018–0.932)
tmig (B) → (A)	0.093 Ma (0.010–0.333)	0.366 Ma (0.010–0.056)	0.221 Ma (0.010–0.456)	0.342 Ma (0.017–1.668)
mig‐rate (A) → (B)	‐	8.2% (0%–25%)	51.3% (0.03%–0.51%)	10.5% (6.5%–39%)
mig‐rate (B) → (A)	20.4% (7.28%–60%)	6.2% (5.6%–24%)	34.0% (0.2%–60%)	10.4% (3.9%–57.6%)
tdiv (A)–(B)/(C)	1.926 Ma (0.618–2.467)	0.592 Ma (0.427–0.684)	0.941 Ma (0.533–1.600)	2.349 Ma (2.028–3.607)
tmig (A)–(B) → (C)	0.096 Ma (0.018–1.722)	0.515 Ma (0.010–0.570)	0.239 Ma (0.154–0.372)	0.342 Ma (0.161–2.141)
tmig (C) → (A)‐(B)	0.632 Ma (0.011–1.487)	0.026 Ma (0.010–0.594)	0.325 Ma (0.051–1.005)	1.989 Ma (0.010–2.388)
mig‐rate (A)‐(B) → (C)	5.7% (0.144%–40.3%)	0.007% (0%–14%)	45.0% (23%–45%)	8.8% (6.2%–60%)
mig‐rate (C) → (A)–(B)	11.2% (0.003%–45%)	2.5% (0%–11%)	25.3% (0.19%–41.82%)	17.3% (0%–60%)
Growth‐rate	0.000001^a^	–	–	–

*Note*: tdiv, Divergence time; tmig, Migration time; mig‐rate, Percentage of Ne that migrated; –, Value not estimated.

^a^ Growth rate for *D. mentalis* clusters. Populations letter as defined in Figure 1.

**FIGURE 6:**
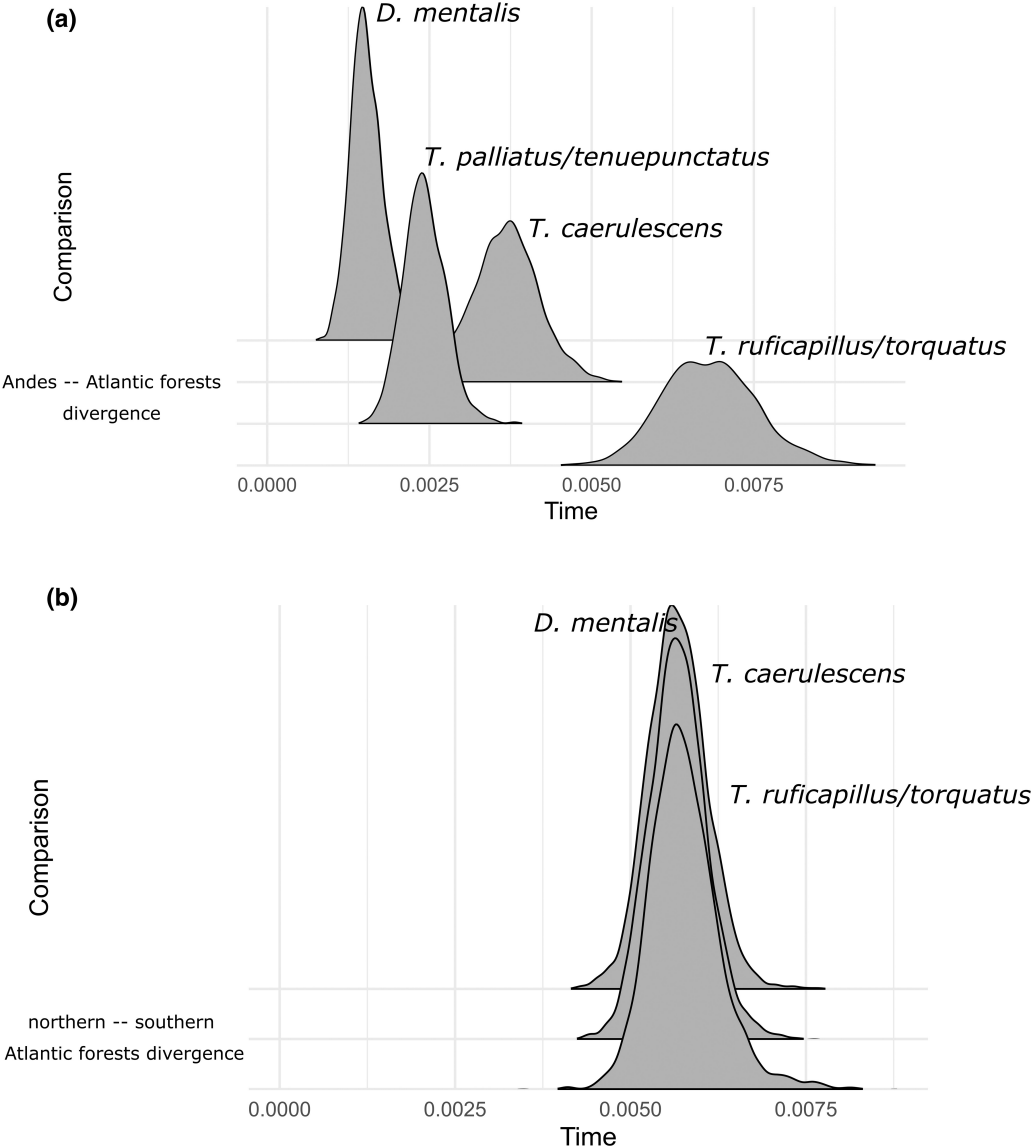
Results of the test of simultaneous divergences across taxa in Ecoevolity. (a) Approximate marginal posterior densities of divergence times (in expected substitutions per site; left present, right past) for each pair in the Andean‐Atlantic Forest clusters. (b) Approximate marginal posterior densities of divergence times (in expected substitutions per site; left present, right past) for each pair in the northern—central southern Atlantic Forest clusters (*T. palliatus*/*tenuepunctatus* complex not included here).

We identified two types of divergence models with *ecoevolity*. For the connection “Andes–Amazonia”, *ecoevolity* estimated four separate divergence events for each taxon. According to demographic models, the *T. ruficapillus*/*torquatus* complex showed the oldest divergence time, whereas the most recent corresponded to *D. mentalis* (Figure 6a). For the divergence between the Northern‐Central and southern Atlantic Forest, the best model depicted a scenario with one simultaneous divergence event across the four taxa regardless of the model. (Figure 6b).

## 4 DISCUSSION

### 4.1 The role of geographical barriers in the circum‐Amazonian distribution pattern

In general, genetic clusters identified here were concordant with known biogeographical units (Bates et al., 1998; Dinerstein et al., 2017; Morrone, 2001; Silva et al., 2004), and the connections among them show certain levels of hybridization and clinal variation, mainly in *T. caerulescens* and *D. mentalis*, a phenomenon found by previous studies (Bolívar‐Leguizamón et al., 2020; Brumfield, 2005; Isler et al., 2005; Todd, 1916; Zimmer & Isler, 2003). Here, geographical barriers play a key role in generating and maintaining population structure (Mayr, 1942; Myers et al., 2019; Pujolar et al., 2022). However, population structure can also arise in the absence of geographical barriers (Nosil, 2008). In the Neotropics, wide river valleys, high mountains like the Andes, and large rivers such as in the Amazon Basin, are known to separate the spatial distribution of numerous taxa (Gonçalves‐Sousa et al., 2022; Hazzi et al., 2018; Hoorn et al., 2010; Wallace, 1854), but the impact of these barriers is not the same for all taxa, being influenced by the organism's vagility, size, and population dynamics (Burney & Brumfield, 2009; Lavinia et al., 2019; Naka & Brumfield, 2018; Smith et al., 2014). When assessing whether geographical barriers prevent gene flow between clusters of individuals, our results suggest that the role of the geographical barriers seems variable across the species studied. The EEMS analysis allowed us to define that these “geographical splits” (areas with migration rates lower than expected) were congruent spatially with the geographical distribution of the genomic clusters recovered. However, most of these geographical barriers seem to have acted as secondary rather than primary barriers because the divergence times estimated for all four taxa do not match the formation of these barriers. Except for *T. caerulescens* (Bolívar‐Leguizamón et al., 2020), physical barriers were not the main promoters of diversification in circum‐Amazonian antbird taxa. Based on our divergence time estimates, the lineage splits within all four species occurred after the rise of the Andes (from the early Miocene to early Pliocene, see Hoorn et al., 2010; Montgomery et al., 2001), or the formation of rivers such as the Paraná or São Francisco (Lanna et al., 2020; Ribeiro et al., 2018). In the best‐fit demographic model, there was no signal of population size variation (except in *D. mentalis* that presented population expansion in the central Atlantic Forest cluster), a scenario that could suggest the isolating effect of physical barriers by preventing clusters from coming into secondary contact. These demographic models also indicate low levels of gene flow between populations separated by specific barriers, such as the São Francisco River for *T. caerulescens* and *D. mentalis*, or the open areas (Cerrado and Chaco) for the *T. ruficapillus*/*torquatus* complex. Nonetheless, these low levels of gene flow between the northern and central‐southern Atlantic Forest clusters (*D. mentalis* and *T. caerulescens*) could be a consequence of a long history of isolation, with very recent contact, as they are not closely related phylogenetically (see Figure 2). These findings are congruent with other studies about barriers as the Andes (see Hazzi et al., 2018; Luebert & Weigend, 2014; Musher et al., 2019; Quintana et al., 2017) and Amazonia (e.g., Dal Vechio et al., 2020; Del‐Rio et al., 2021; Naka & Brumfield, 2018; Nazareno et al., 2017; Pirani et al., 2019). Research on the importance of barriers to the diversification of non‐Amazonian taxa has increased in recent years, highlighting the importance of barriers in driving and maintaining population differentiation in South American biomes (Baranzelli et al., 2020; Cáceres, 2007; Giudicelli et al., 2022; Kopuchian et al., 2020; Nascimento et al., 2013).

### 4.2 Biogeographical and phylogenetic relationships of circum‐Amazonian birds

Phylogenetic relationships within circum‐Amazonian birds revealed some commonalities. The northern and the central‐southern Atlantic Forest groups have been identified previously in toads and birds (Batalha‐Filho, Irestedt, et al., 2013; Bocalini et al., 2021; Thomé et al., 2010). However, we found that the species with a complete distribution in the Atlantic Forest region (*D. mentalis* and *T. caerulescens*) do not have a sister relationship between these two clades, contrary to the reported by other studies (Bocalini et al., 2021; D'horta et al., 2011; Franco et al., 2017). In fact, for *D. mentalis*, the *emiliae* clade (northern Atlantic Forest + eastern Amazonia) was more closely related to the *affinis* clade (SDTFs south of Amazonia) than to the central‐southern Atlantic Forest group. These results are congruent with studies of taxa with Atlantic Forest distribution, whereby the northern portion of this region does not exhibit a sister relationship with the central‐southern areas (Carvalho et al., 2017; Lima et al., 2018; Machado et al., 2018).

Close phylogenetic and biogeographical relationships between the Andes and the eastern portion of the circum‐Amazonian distribution were prominent. For instance, populations of *T. caerulescens* in the Central Andes were found to be sisters to populations in the Atlantic Forest, and Andean populations of *D. mentalis* and *T. palliatus* were found to be closely related to populations in SDTFs south of Amazonia, coinciding with patterns from other groups (Cadena et al., 2019; Lavinia et al., 2019; Trujillo‐Arias et al., 2017). The inclusion of the Tepuis populations into the Andean clade in *D. mentalis* is also recovered by other analyses. Similar results were found by Borges et al. (2018), who concluded that the Pantepui region is a biogeographical unit separated from the Andes, but with probable past interconnections with the northern Andes (Bonaccorso & Guayasamin, 2013).

A special case is the *T. ruficapillus*/*torquatus* complex systematics; where the *T. ruficapillus* from the Atlantic Forest is closest to the *T. torquatus* (with a contact zone and high level of hybridization between them) than to Andean *T. ruficapillus* (Figure 2; Figure S7). Additionally, the best‐fit demographic model estimated the same divergence times for the complex (Figure 6). This pattern could be a consequence of (a) the geographical separation between the Andes and Atlantic Forest populations of *T. ruficapillus* may be due to geographical or climatic factors, generating an increase in the genetic differentiation; and (b) the hybridization of the Atlantic Forest population of *T. ruficapillus* with the open‐areas *T. torquatus* populations. In their work about the genus *Thamnophilus*, Brumfield and Edwards (2007) found that in the group called the “barred clade”, the *ruficapillus*/*torquatus* complex was indeed a monophyletic group, with a common ancestor that could be a highland‐restricted or a lowlands‐to‐highlands taxon. A possible scenario is that of “lowlands‐to‐highlands” origin from the southern Atlantic Forest region moving west to the central Andes (Andean *ruficapillus* populations) and north to the forested areas in central/northern Brazil, with posterior isolation of the morphologically differentiated *T. torquatus*. A recent secondary contact could explain the hybrid zone between the southern *T. ruficapillus* and the *T. torquatus* populations (Brumfield & Edwards, 2007).

### 4.3 Historical demography

Our mitochondrial divergence time estimates are compatible with a Quaternary rise in the genetic structuring of these species, as has been found in other thamnophilids (Bolívar‐Leguizamón et al., 2020; Choueri et al., 2017; Ribas et al., 2018). Divergence times estimates based on demographic modeling of the UCE SNPs dataset were highly dependent on other model parameters—especially the population size, *Ne*. Additionally, because UCEs do not have a single substitution rate, a comparison of divergence time estimates obtained with mtDNA and UCEs is difficult. Mito‐nuclear discordance has been documented in multiple taxa, where the phylogenetic signals of mtDNA and nuDNA are not equal and the topologies recovered from each genetic source are discordant (Rheindt & Edwards, 2011; Toews & Brelsford, 2012). Multiple evolutionary and ecological explanations for this phenomenon have been proposed (e.g., incomplete lineage sorting, adaptive introgression of mtDNA, asymmetric mate choice, among others), but testing these phenomena was beyond the scope of our study. Here, we tried to minimize these problems by testing multiple *Ne* values for the initial models and used the substitution rate proposed by Nadachowska‐Brzyska et al. (2015).

Our demographic models suggested that the four antbird groups share a similar demographic history. Besides the Pleistocene origin, all groups showed evidence of bidirectional migration and absence of events of populational expansion/retraction, with only *D. mentalis* showing populational expansion in the central‐southern Atlantic Forest (Table 1, Figure 4a). The best‐fit model(s) is consistent with the initial presence of a great forested region, subdivided into interconnected refugia/forested spots during the climatic fluctuations in the Pliocene–Pleistocene (Refugia Hypothesis, see Haffer, 1969 and Vanzolini & Williams, 1970), allowing partial isolations and intermittent gene flow among populations, resulting in the origin of new lineages (Figures 4 and 5). Several authors used the climatic fluctuations during the Cenozoic as an explanation for the diversification of lineages of multiple groups for the Amazonian (Pupim et al., 2019; Richardson et al., 2001; Silva et al., 2018), and non‐Amazonian biotas (García‐Vázquez et al., 2017; Madriñán et al., 2013; Mascarenhas et al., 2019; Pérez‐Escobar et al., 2017; Thomaz et al., 2015). However, it is important to clarify that the nature and intensity of these climatic oscillations in the diversification of the taxa could be different for each species. For instance, despite the four groups having a circum‐Amazonian distribution, they do not have the same type of forested habitats (humid vs. dry forests), and a changing climate of humid‐warm and dry‐cold cycles during the Quaternary would affect them individually (Silva, 1994).

We found evidence of asynchronous divergences for the Andes‐Atlantic Forest division and synchronous events of divergence between the northern and the south‐central Atlantic Forest. The *T. ruficapillus*/*torquatus* complex showed a more ancient divergence when compared to the other three species complexes (Figure 5 and 6; Figures S3 and S4). These idiosyncratic histories can be explained by the different responses of each group to climatic fluctuations during the Quaternary. Examples of asynchronous divergence times across co‐distributed taxa have been reported in the literature (Bocalini et al., 2021, 2023; Kopuchian et al., 2020; Lavinia et al., 2019; Leaché et al., 2020; Oswald et al., 2017), suggesting that this phenomenon is a common scenario across codistributed taxa. Our results for the Andes‐Atlantic Forest split contradict the premise that closely related codistributed lineages should have similar responses to the same factors (Papadopoulou & Knowles, 2016). On the other hand, our results showed a congruent event of divergence for the species that shared a split between the northern and central/southern Atlantic Forest clusters (early Pleistocene). We suggest that these shared divergences are a response to the climatic events during the Quaternary. Codivergence is expected in groups with similar evolutionary characteristics, like substitution rates, reproduction strategies, habitats, and ecological requirements, among others (Leaché et al., 2020). A strength of our work is the phylogenetic proximity of the species analyzed: the *T. palliatus*/*tenuepunctatus* and the *T. ruficapillus*/*torquatus* complexes belong to a “barred clade”, while the *T. caerulescens* into the “solid clade”, and *D. mentalis* as close to the genus (Brumfield & Edwards, 2007; Harvey et al., 2020). This evolutionary link minimized the noise produced in analyses where distant codistributed species are analyzed together. An example of simultaneous divergence events as a consequence of climate change can be the Great American Biotic Interchange (GABI, see Webb, 1991). In an analysis using molecular and paleontological data, Bacon et al. (2016) concluded that climatic and environmental changes were the most likely trigger for the GABI in mammals and estimated a simultaneous time of diversification approximately between the Late Pliocene to early Pleistocene (see Woodburne, 2010).

The different results in the two *ecoevolity* analyses (Andes‐Atlantic Forest vs. northern vs. central/southern Atlantic Forest splits) using the same taxa seem not intuitive. One possible explanation is the differing effect of the glacial cycles along the circum‐Amazonian region. Paleoclimate analyses have inferred that the climatic variations through the Quaternary were varying in their areas of influence (see Baker et al., 2020, for the Amazonian region). The climatic fluctuations may have affected differently the Atlantic Forest regions than in the Andes‐Atlantic Forest connection region. However, a revision about the Paleoenvironmental evolution of Southern South America, Ortiz‐Jaureguizar and Cladera (2006) stated that the cold and dry climates during the Quaternary were similar in all the affected areas, while more recent works claim that local climatic conditions were also important for the evolution of the biota during the Quaternary (Cabanne et al., 2016; Ledo & Colli, 2017). Another interesting point is the topographic differences in the covered areas by the two splits (the divergences); the “wide connection” between the Andes‐Atlantic Forest has a diverse number of habitats impulsed by a heterogeneous topography that includes dry forests (Chiquitano), the Chacoan ecoregions (the dry and humid Chaco), humid forested areas (southern Atlantic Forest and montane forests in the central Andes), and some open areas as flooded grasslands and savannas (Beni Savanna and Pantanal) (see Dinerstein et al., 2017). This diversity in habitats and the influence of global glacial cycles during the Pleistocene (promoting the existence of isolated forested patches in these areas) might have facilitated numerous biogeographical connections between the Andes and the Atlantic Forest areas. These multiple connections could have allowed the emergence of this pattern of idiosyncratic divergence or migration, whereas distinct populations have diverged or migrated at various intervals mediated by climate change (Kopuchian et al., 2020; Trujillo‐Arias et al., 2017, 2020). On the other hand, the northern‐central Atlantic Forest split covers a restricted area, also very diverse in habitats (mainly due to broad latitudinal and altitudinal ranges), but geographically narrower, mainly in the northern‐central Atlantic Forest division (Lundberg et al., 1998; Ribeiro et al., 2009). This lack of geographical extension could increase the probability of synchronic events of divergence in the taxa analyzed here, despite the great disturbance forces created by the climatic oscillations during the Quaternary (plus the altitudinal factor) that affected this biome (Carnaval et al., 2009, 2014; Thom, Smith, et al., 2020). In the split between the “Andes‐Atlantic Forest” populations in *D. mentalis* (see Figure 2) the divergence between the northern (*emiliae* group) and the “*affinis* + Andean” clades was posterior (0.63 ma) to the divergence between them with the central/southern Atlantic Forest cluster (*mentalis* group, 1.92 ma), suggesting early‐strong isolation of the *mentalis* group from the others populations, maybe due to effects of glacial cycles during the Quaternary and local factors (geomorphological properties, altitude) in open areas as Cerrado (Brusquetti et al., 2023; Ledru et al., 2006; Salgado‐Labouriau, 1994; van der Hammen & Hooghiemstra, 2000). Posteriorly, the remnants of gallery/dry forested areas in Cerrado were further isolated in the last glaciation events (e.g., LGM, see Figure 4a). Finally, Oaks et al. (2020) suggested that *ecoevolity* could be sensible to events of migration with significant gene flow, whereas secondary migration events with high levels of gene flow could be confused as real divergence events by the algorithm. The sNMF and *momi2* results show admixture and migrations between the northern and the central/southern Atlantic Forest regions for *D. mentalis*, *T. Caerulescens*, and the *T. ruficapillus*/*torquatus* complex. We tried to reduce the impact of this phenomenon eliminating hybrid individuals in *momi2*. Nevertheless, the results of *ecoevolity* for the northern‐central/southern Atlantic Forest split showed consistent “parallel” demographic events (Figure 6), backing the possibility of shared responses to the ecological/geological processes affecting these ecoregions, as climatic oscillations and the geographical extend of the split.

Our results suggest a “southeastern” diversification, with the early isolation of populations from the Atlantic Forest to the southwest to the central Andes, and a subsequent differentiation among them (in *T. caerulescens* and *D. mentalis*). In the *T. palliatus*/*tenuepunctatus* and the *T. ruficapillus*/*torquatus* complexes, an early diversification could take place in the central Andes. This scenario could be congruent with the stated by Brumfield and Edwards (2007), who suggested that the Andean populations (at least for *T. caerulescens*) represent a secondary invasion of the species via a forest bridge between southeastern Brazil and the central Andes. This approach was proposed initially by Chapman (1926), who suggested the presence of a former corridor of humid forest between the humid forests of southeastern Brazil and the humid slopes of the Andes could explain the origin of some of the Andean foothill taxa. Here, the *D. mentalis* and the *T. caerulescens* seem to show this pattern. On the other hand, this statement must be taken with caution, since the best models for the species did not include bottlenecks; a common indication of secondary invasion. Multiple authors studied these possible connections between the Andes and the Atlantic Forest (Batalha‐Filho, Fjeldså, et al., 2013; Trujillo‐Arias et al., 2017, 2020). In a work about the biotic interchange between the Amazonian and the Atlantic Forest regions, Ledo and Colli (2017) concluded that a southern route (southeastern Atlantic Forest ←→ western Amazonia) was the most probable scenario to explain the connection between these regions, and this same scenario can be framed into the central Andes ←→ southern Atlantic Forest connections. Yet, the exact path of this diversification appears not to be the same across all groups. Nevertheless, the majority of studies align in suggesting that it occurred during the Pliocene–Pleistocene period, correlated with the climatic fluctuations of these epochs (Cabanne et al., 2019; Camps et al., 2018). We found general congruence among the populational structure, phylogenetic relationships, and demographic histories of the taxa analyzed. We defined two main units: the Andean and the eastern Brazilian forested (mainly the Atlantic Forest) phylogeographical regions, disjointed complete or partially a southern interconnection (southern Atlantic Forest ←→ central Andes). Contact zones among clusters included individuals with considerable levels of admixed genotypes, which indicates current and historical hybridization among populations, mainly in the Andes‐Atlantic Forest connection (Bolívar‐Leguizamón et al., 2020; Brumfield & Edwards, 2007). Demographic histories of the four taxa seem to be a product of a recent diversification with climatic fluctuations throughout the Pliocene–Quaternary as its main influence, fitting into a Forest Refugia context. Our analyses also inferred asynchronous divergences in the connection Andes‐Atlantic Forest, while the northern‐central/southern Atlantic Forest regions seem to represent a simultaneous divergence event. This study is an analysis of the circum‐Amazonian distributional pattern, incorporating genomic data for four complexes of passerine birds. The results provide valuable insights into the evolutionary and ecological processes that have shaped this distribution pattern, enhancing our understanding of it. However, some key regions of the distribution were not sampled or the collected material was insufficient, mainly from the northern Andes and the Tepuis, limiting the analysis of evolutionary scenarios that test a northern interconnection among northern Andes, Tepuis, and the northern Atlantic Forest regions. In the same way, alternative scenarios that explain the circum‐Amazonian distribution pattern were not tested in this work. For instance, competition relationships that could limit the distributions of the groups across large spatial and temporal scales (Pigot & Tobias, 2013; Price & Kirkpatrick, 2009; Rabosky, 2013; Terborgh & Weske, 1975; Weir & Price, 2011), or processes of species interaction constraints such as competitive exclusion. In this case, species exhibiting a circum‐Amazonian distribution would have their ranges limited by the presence of closely related, ecologically similar species in Amazonia.

## 5 CONCLUSIONS

This study found similarities at populational, phylogenetic, and evolutionary levels among four taxonomic groups of passerine birds with a circum‐Amazonian distribution. We show evidence that taxa with circum‐Amazonian distribution are formed by two main phylogeographical clusters: (1) Andes, often extending into Central America and the Tepuis; and (2) the remaining of their distribution. Also, the northern Atlantic Forest and the central‐southern Atlantic Forest are separated phylogeographical units that are not necessarily closed related. We concluded that the presence of Forest Refugia resulted from climatic oscillations during the Pleistocene was the primary driver in the diversification of the taxa. This phenomenon also facilitated subsequent migration events among their populations along environmental gradients. However, the inclusion of biotic factors must be tested to pinpoint critical variables driving distribution patterns and allowing migration events. Additionally, the tempo of these divergences was synchronic for the Atlantic Forest populations and asynchronous for the Andes‐Atlantic Forest/SDTFs divergences. Our phylogenetic analyses also suggest that the *T. ruficapillus*/*torquatus* complex needs taxonomic revision since *T. ruficapillus* from the southern Atlantic Forest is more closely related to *T. torquatus* than to *T. ruficapillus* in the Andes and Chaco. Future studies about the circum‐Amazonian distribution must address the role of biotic interactions in driving the distributional patterns, as well as the analysis of other taxonomic groups and the inclusion of ecological and climatic data.

## AUTHOR CONTRIBUTIONS

**Sergio D. Bolívar Leguizamón:** Conceptualization (equal); data curation (equal); formal analysis (equal); funding acquisition (equal); investigation (equal); methodology (lead); project administration (equal); resources (equal); software (lead); supervision (equal); validation (equal); visualization (equal); writing – original draft (lead); writing – review and editing (equal). **Fernanda Bocalini:** Conceptualization (equal); data curation (equal); formal analysis (equal); funding acquisition (equal); investigation (equal); methodology (equal); project administration (equal); resources (equal); software (equal); supervision (equal); validation (equal); visualization (equal); writing – original draft (equal); writing – review and editing (equal). **Luís F. Silveira:** Conceptualization (equal); data curation (equal); formal analysis (equal); funding acquisition (equal); investigation (equal); methodology (equal); project administration (equal); resources (equal); software (equal); supervision (equal); validation (equal); visualization (equal); writing – original draft (equal); writing – review and editing (equal). **Gustavo A. Bravo:** Conceptualization (equal); data curation (equal); formal analysis (equal); funding acquisition (equal); investigation (equal); methodology (equal); project administration (equal); resources (equal); software (equal); supervision (equal); validation (equal); visualization (equal); writing – original draft (equal); writing – review and editing (equal).

## FUNDING INFORMATION

Financial support was provided by the São Paulo Research Foundation—FAPESP (2015/16092‐7 and 2022/16202‐0 to SDB–L; 2012‐23852‐0 to GAB; 2017/23548‐2 to LFS; and 2020/16065‐8 to FB), National Science Foundation—NSF (DEB‐1011435 to GAB), and Brazilian Research Council—CNPq (457974‐2014‐1 and 308337/2019‐0 to GAB and LFS). SDB‐L and FB acknowledge financial support from the Coordination for the Improvement of Higher Education Personnel—CAPES and the Frank M. Chapman Memorial Fund from the American Museum of Natural History—AMNH (2016).

## Supporting information


Figure S1.

Figure S2.

Figure S3.

Figure S4.

Figure S5.

Figure S6.

Figure S7.

Table S1.

Table S2.

Table S3.

Table S4.

Table S5.

Table S6.

Table S7.

Table S8.

Table S9.

Table S10.

Table S11.

Data S1.

## Data Availability

UCE raw read data are available on NCBI SRA (BioProject PRJNA1064987). VCF files, UCE sequence alignments, and scripts of the models tested are available at https://github.com/SergioB1983.
