# Supplementary figures and images for: The role of biogeographical barriers on the historical dynamics of passerine birds with a circum‐Amazonian distribution
Source: Ecol Evol. 2024 Mar 6;14(3):e10860. doi: 10.1002/ece3.10860 (PMC10915597; doi:10.1002/ece3.10860)

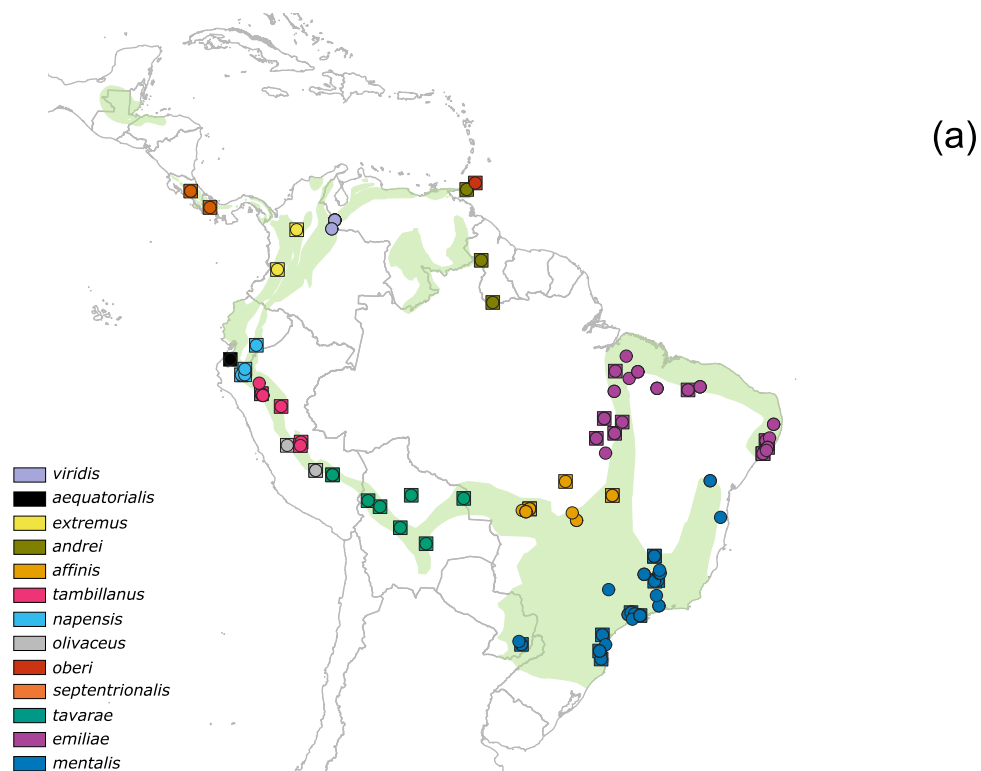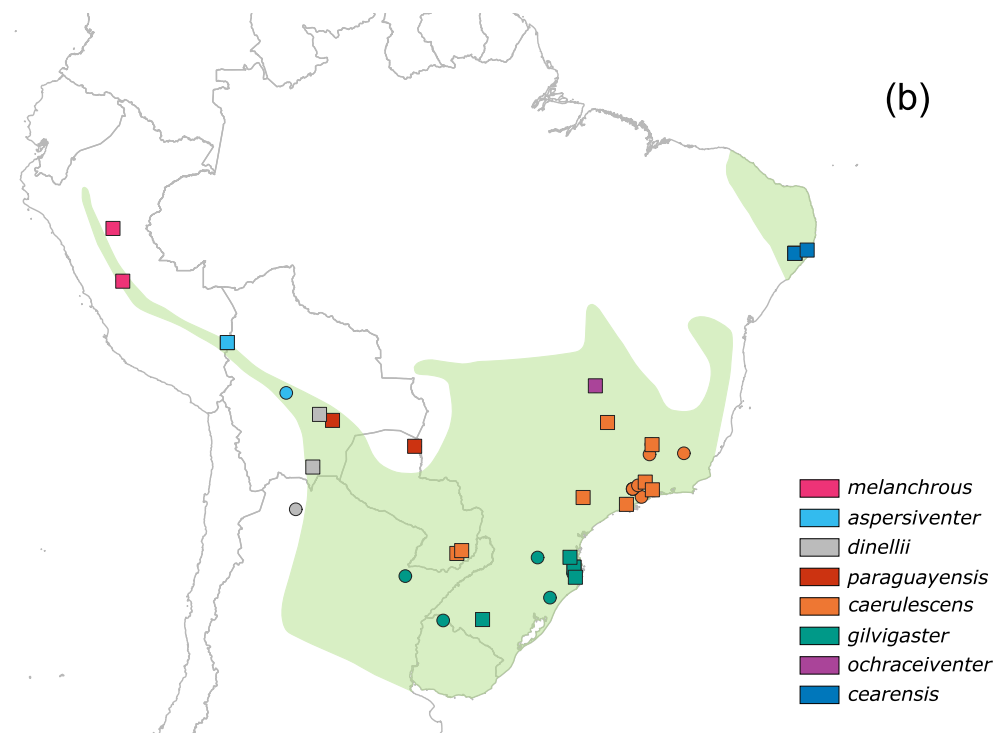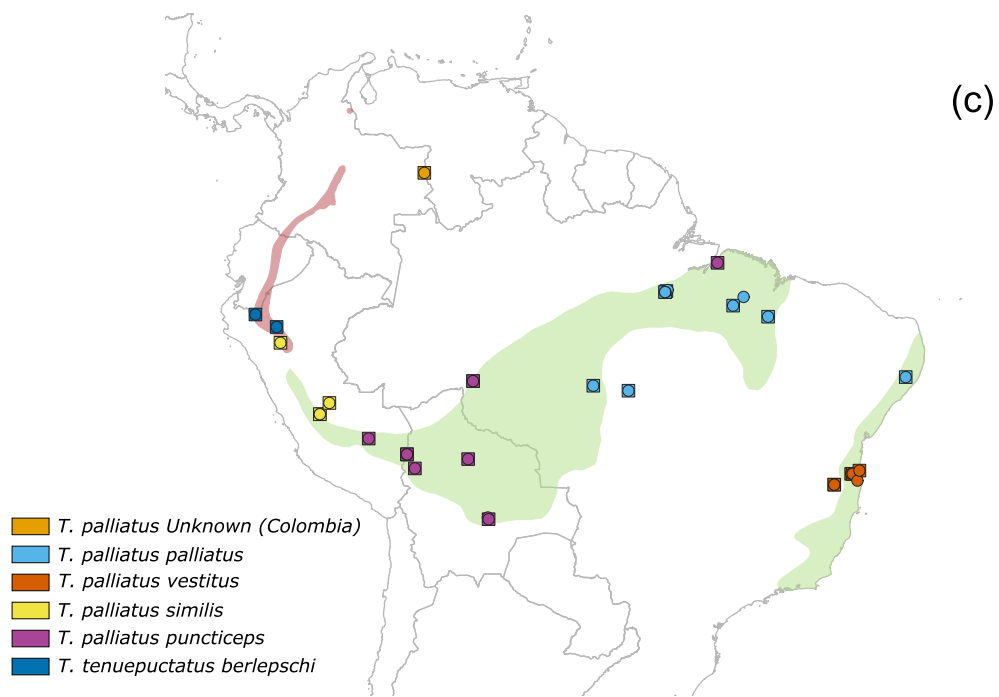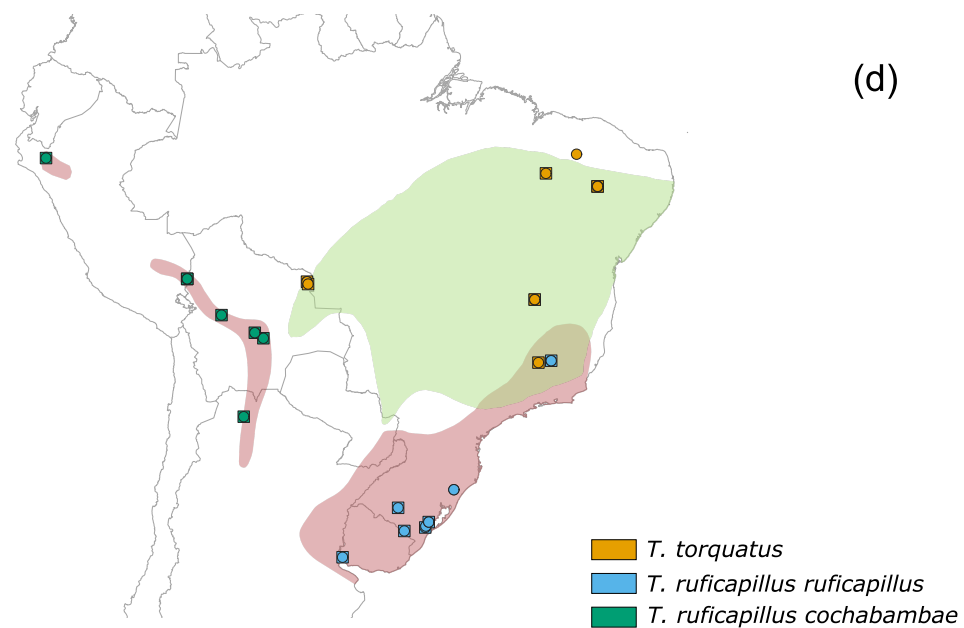

Supplement: Supplementary file 1 — Figure S1. [file ECE3-14-e10860-s010.pdf]

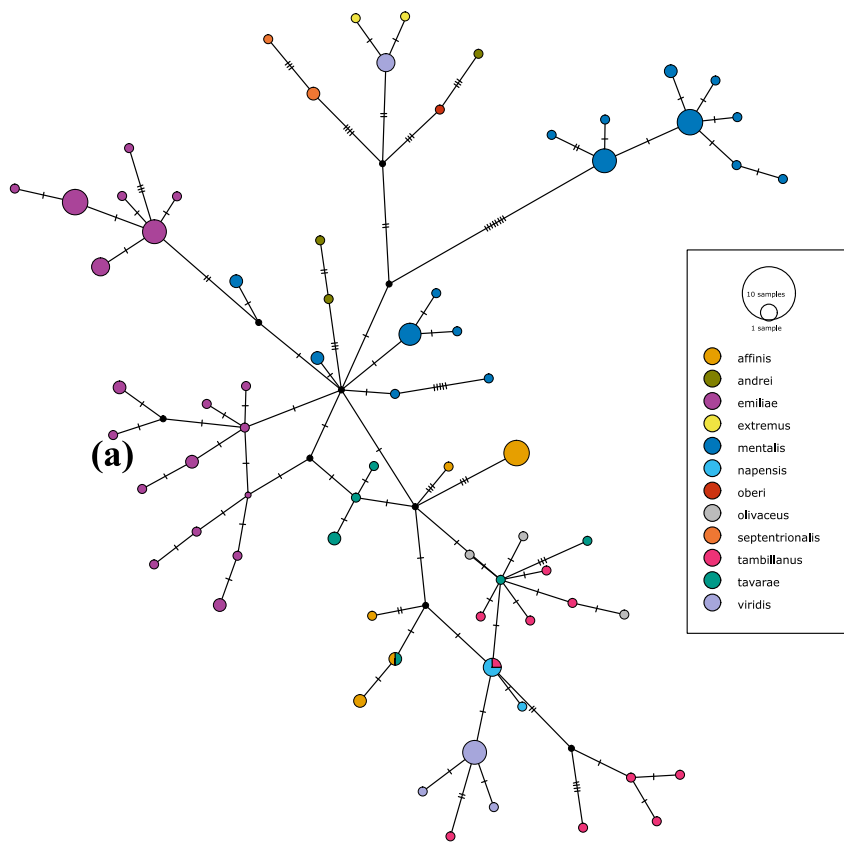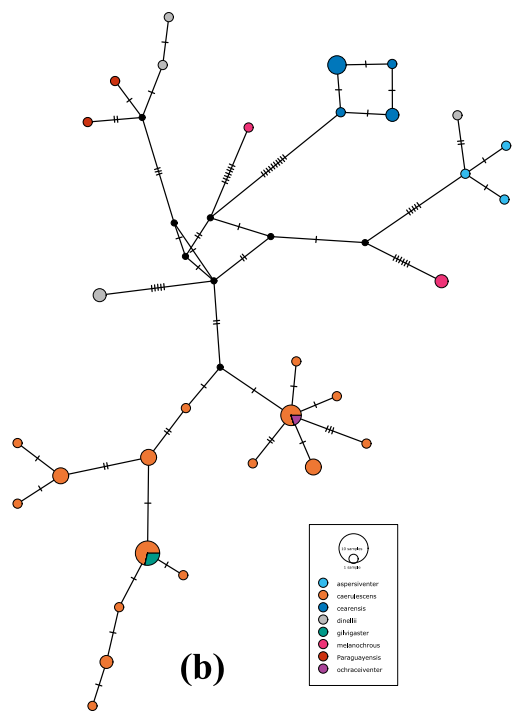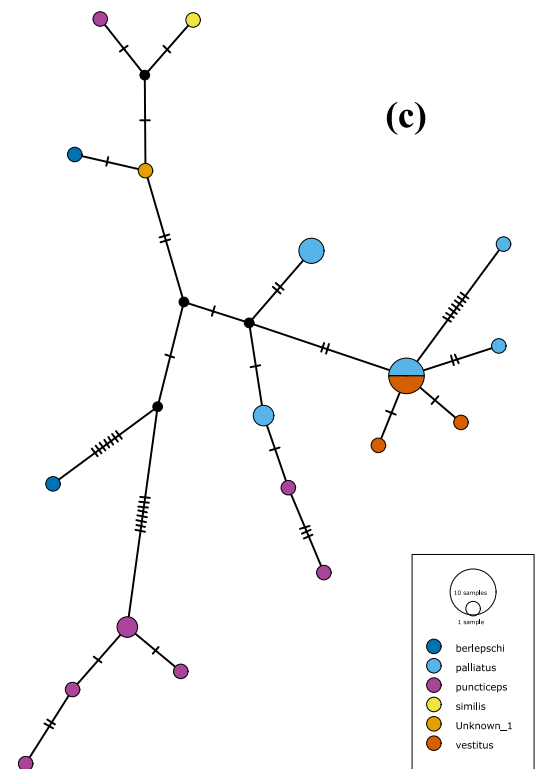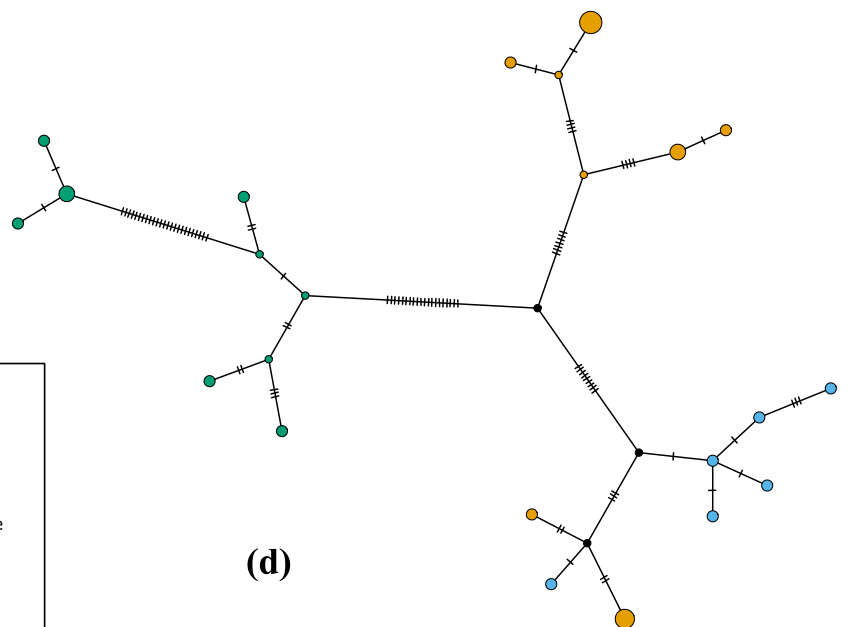

Supplement: Supplementary file 2 — Figure S2. [file ECE3-14-e10860-s003.pdf]

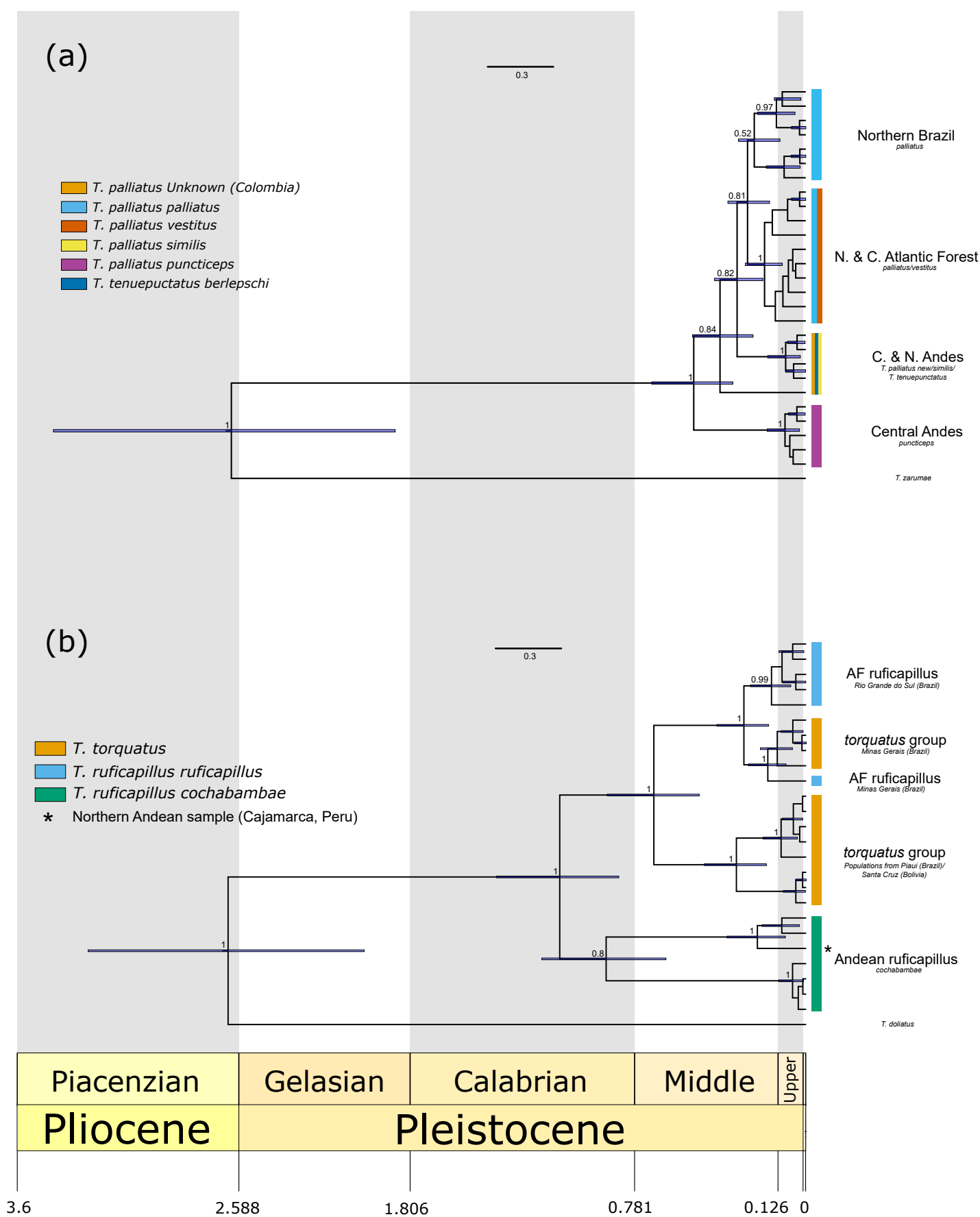

Supplement: Supplementary file 4 — Figure S4. [file ECE3-14-e10860-s008.pdf]

**(a)**

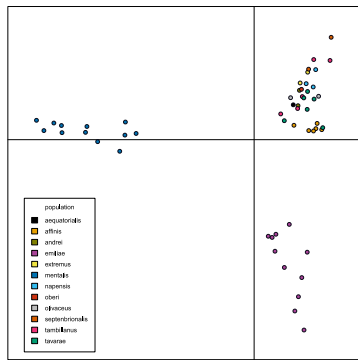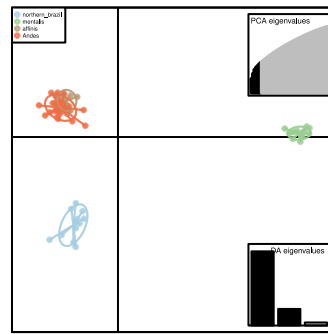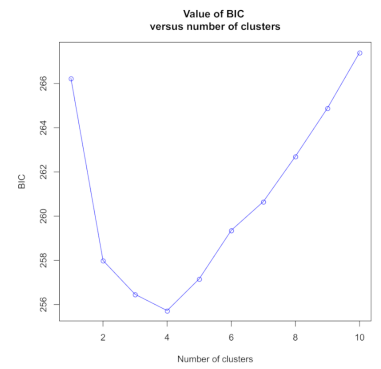

(b)

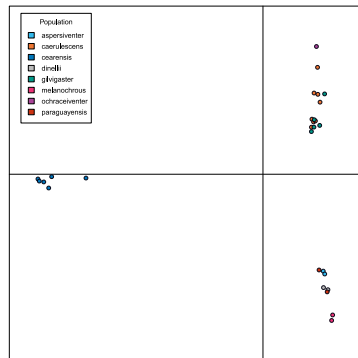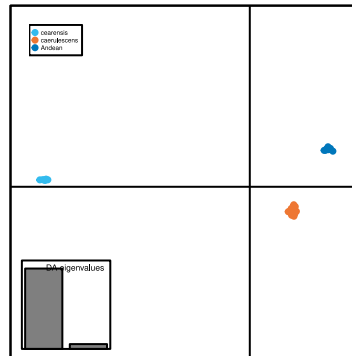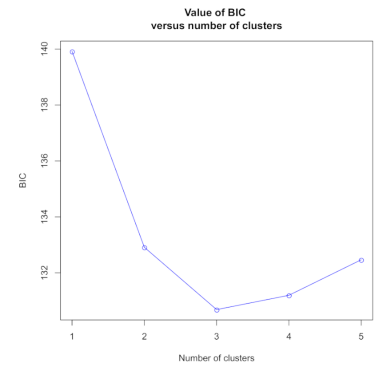

**(c)**

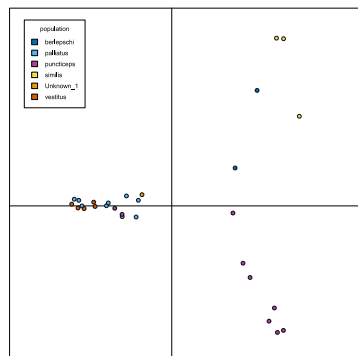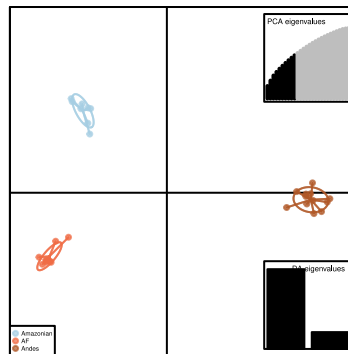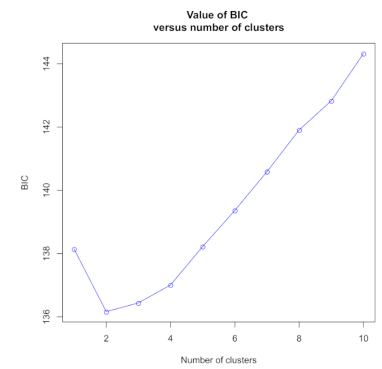

**(d)**

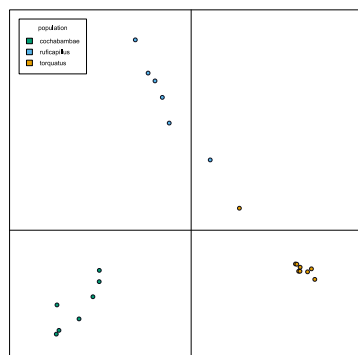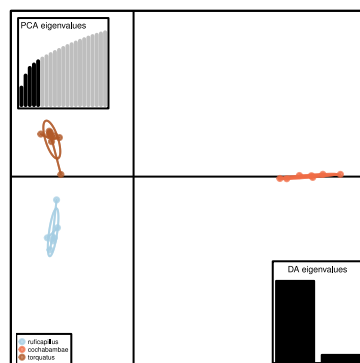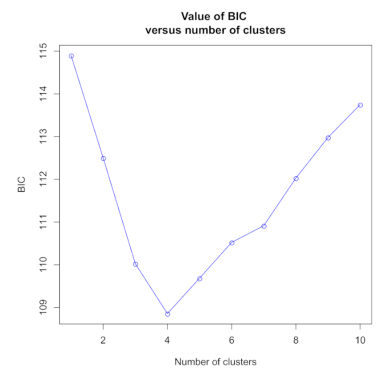

Supplement: Supplementary file 5 — Figure S5. [file ECE3-14-e10860-s013.pdf]

(a)

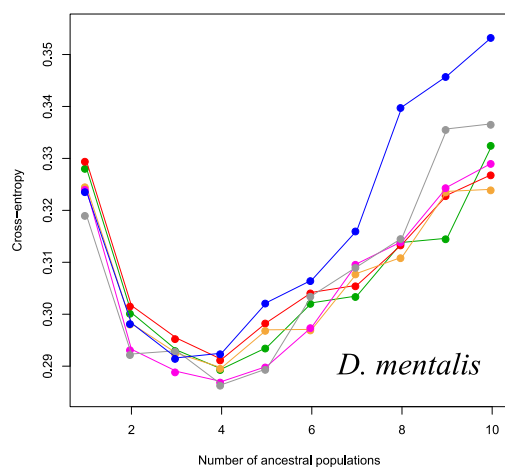

(b)

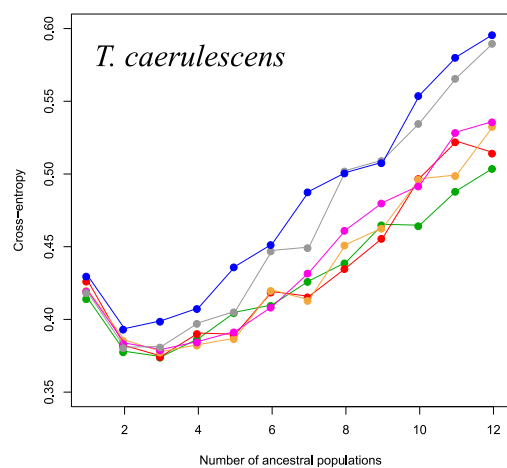

(c)

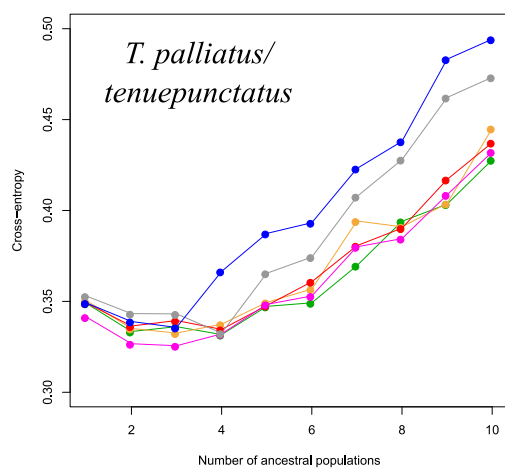

(d)

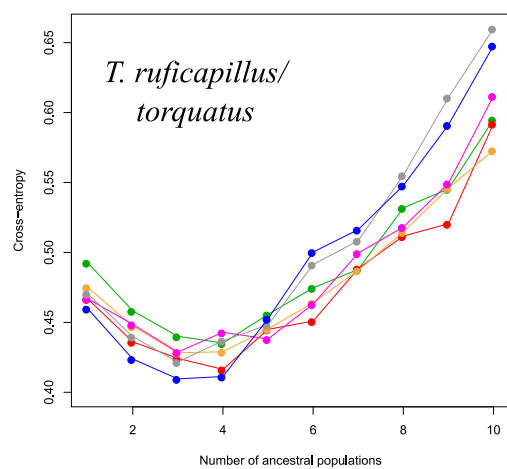

Supplement: Supplementary file 6 — Figure S6. [file ECE3-14-e10860-s016.pdf]
