# Supplementary material for: The role of biogeographical barriers on the historical dynamics of passerine birds with a circum‐Amazonian distribution
Source: Ecol Evol. 2024 Mar 6;14(3):e10860. doi: 10.1002/ece3.10860 (PMC10915597; doi:10.1002/ece3.10860)
Supplement: Supplementary file 3 — Figure S3. [file ECE3-14-e10860-s004.pdf]

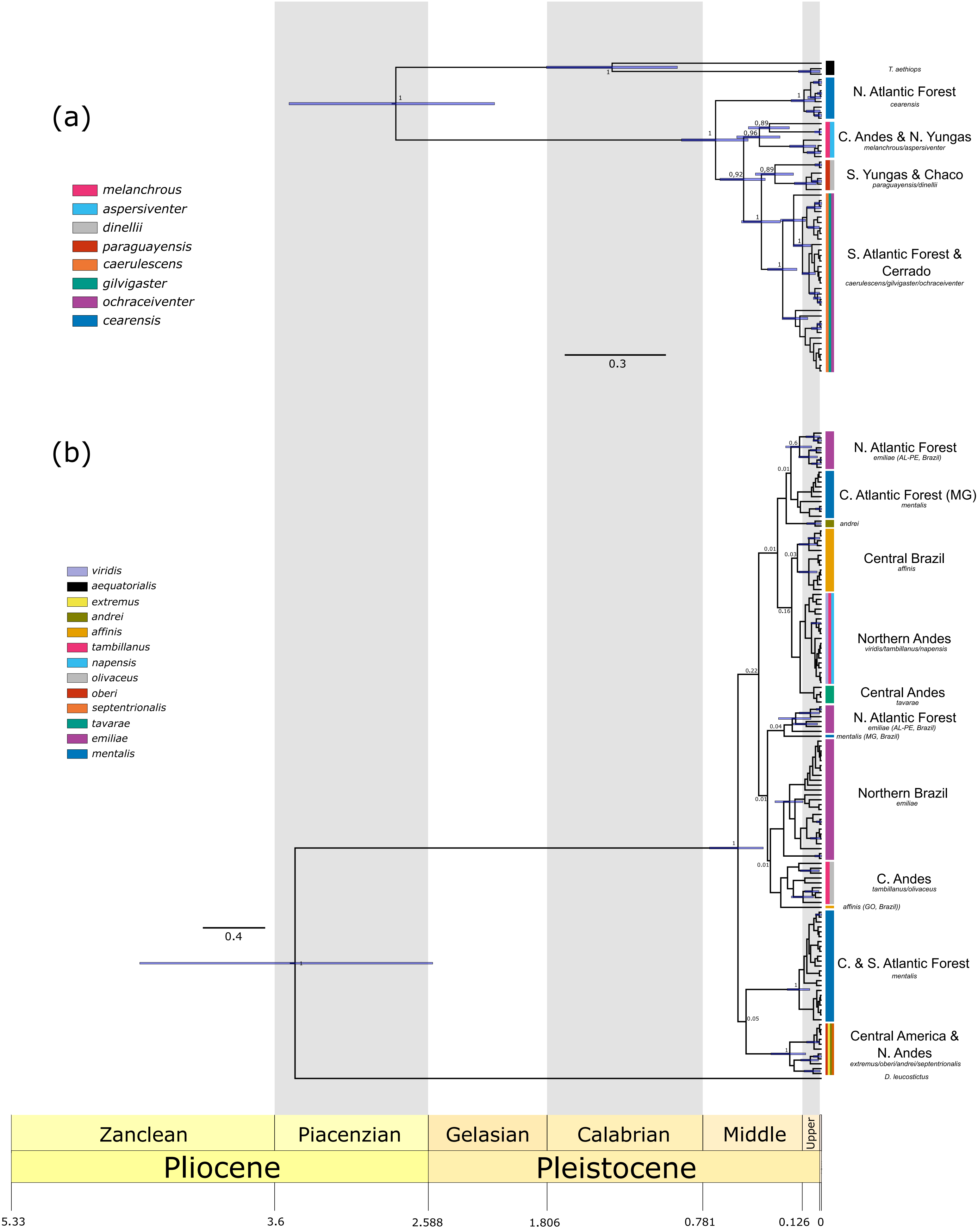

(a)
melanchrous
aspersiventer
dinellii
paraguayensis
caerulescens
gilvigaster
ochraceiventer
cearensis
T. aethiops
N. Atlantic Forest
cearensis
C. Andes & N. Yungas
melanchrous/aspersiventer
S. Yungas & Chaco
paraguayensis/dinellii
S. Atlantic Forest & Cerrado
caerulescens/gilvigaster/ochraceiventer
0.3
(b)
viridis
aequatorialis
extremus
andrei
affinis
tambillanus
napensis
olivaceus
oberi
septentrionalis
tavarae
emiliae
mentalis
N. Atlantic Forest
emiliae (AL-PE, Brazil)
C. Atlantic Forest (MG)
mentalis
andrei
Central Brazil
affinis
Northern Andes
viridis/tambillanus/napensis
Central Andes
tavarae
N. Atlantic Forest
emiliae (AL-PE, Brazil)
mentalis (MG, Brazil)
Northern Brazil
emiliae
C. Andes
tambillanus/olivaceus
affinis (GO, Brazil))
C. & S. Atlantic Forest
mentalis
Central America & N. Andes
extremus/oberi/andrei/septentrionalis
D. leucostictus
0.4
Zanclean
Piacenzian
Gelasian
Calabrian
Middle
Upper
Pliocene
Pleistocene
5.33
3.6
2.588
1.806
0.781
0.126
0
