# Supplementary material for: The role of biogeographical barriers on the historical dynamics of passerine birds with a circum‐Amazonian distribution
Source: Ecol Evol. 2024 Mar 6;14(3):e10860. doi: 10.1002/ece3.10860 (PMC10915597; doi:10.1002/ece3.10860)
Supplement: Supplementary file 19 — Data S1. [file ECE3-14-e10860-s012.docx]

**Methods (Supplementary material)**

*Species trees*

We ran three million iterations in SNAPP in two runs (Bryant, Bouckaert, Felsenstein, Rosenberg, & RoyChoudhury, 2012) to identify the relationships among the clusters recovered in the PCA and sNMF analyses for each taxon.

For *D. mentalis*, we run two configurations based in the structure analyses. The first included three clades (1. northern Atlantic Forest, and eastern Amazonian Forests; 2. central and southern Atlantic Forest; 3. forested areas of eastern Cerrado, Andes, Tepuis, and Central America), and *D. leucostictus*. The second analysis included four clades (1. northern Atlantic Forest, and eastern Amazonian Forests; 2. central and southern Atlantic Forest; 3. forested areas of eastern Cerrado; 4. the Andes + Tepuis, and Central America) and *D. leucostictus*,

We divided *T. caerulescens* into three distinct clusters (1. northern Atlantic Forest; 2. southeastern Cerrado, central, and southern Atlantic Forest; 3. Chaco + central Andes) and *T. aethiops*.

For the *T. palliatus/tenuepunctatus* complex, we run two configurations based in PCA/sNMF analyses. The first analysis included two clades (1. *T. palliatus* and 2. *T. tenuepunctatus* clades), and *T. multistriatus*/*zarumae*. A second run with three clades (1. Atlantic Forest + forested areas of southeastern Amazon; 2. Bolivia to central Peru; 3. northern Peru), and the outgroup.

For the *T. ruficapillus/torquatus* complex, we run two configurations based in the structure analyses. The first analysis included two clades (1. *T. ruficapillus*; 2. *T. torquatus* clades), and *T. doliatus*, and a second run with three clusters (1. *T. torquatus*; 2. andean *T. ruficapillus*; 3. *T. ruficapillus* from central/southern Atlantic Forest), and *T. doliatus*.

**References**

Bryant, D., Bouckaert, R., Felsenstein, J., Rosenberg, N. A., & RoyChoudhury, A. (2012). Inferring Species Trees Directly from Biallelic Genetic Markers: Bypassing Gene Trees in a Full Coalescent Analysis. *Molecular Biology and Evolution*, *29*(8), 1917–1932. doi: 10.1093/molbev/mss086
