# Supplementary material for: The role of biogeographical barriers on the historical dynamics of passerine birds with a circum‐Amazonian distribution
Source: Ecol Evol. 2024 Mar 6;14(3):e10860. doi: 10.1002/ece3.10860 (PMC10915597; doi:10.1002/ece3.10860)
Supplement: Supplementary file 7 — Figure S7. [file ECE3-14-e10860-s009.pdf]

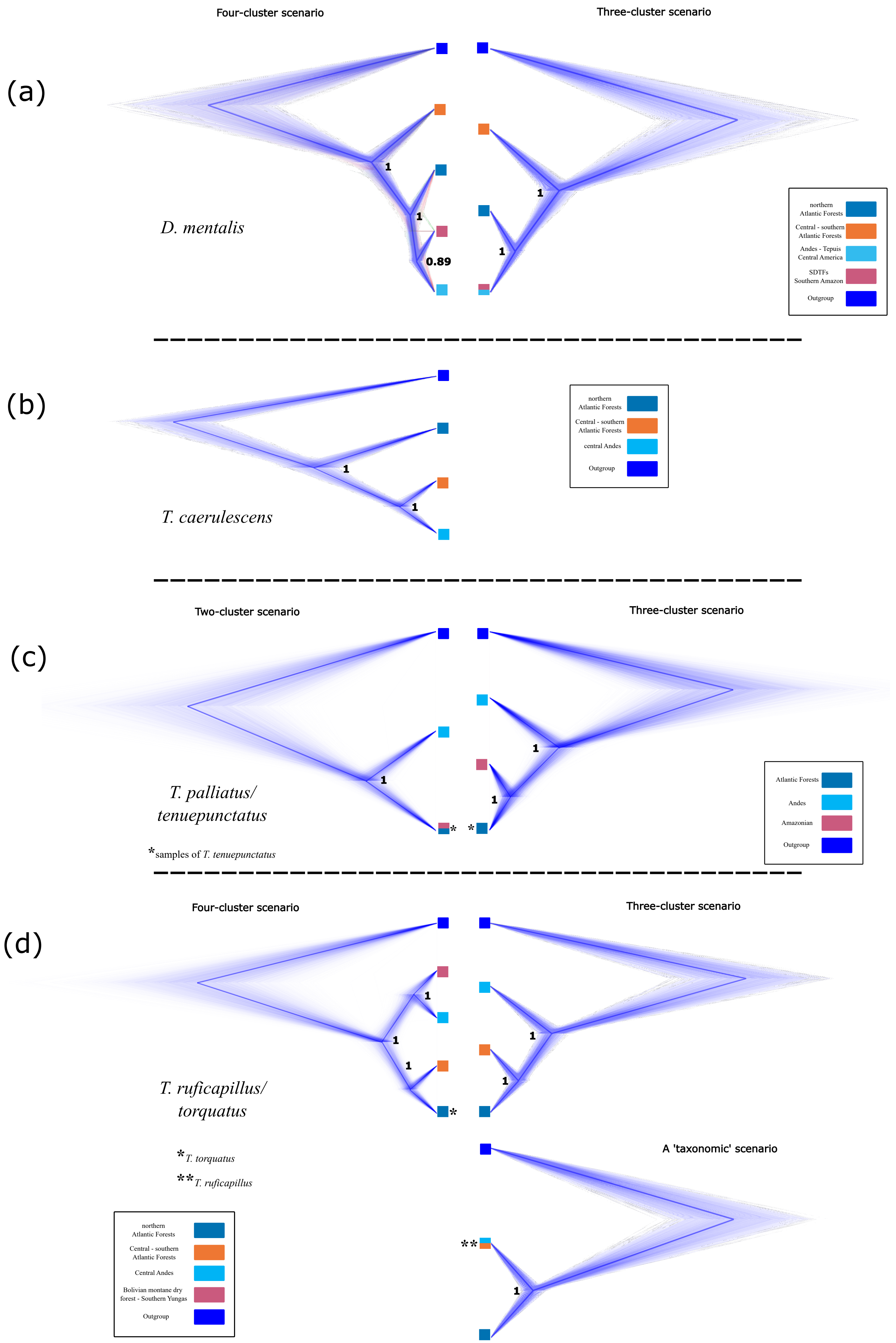
Four-cluster scenario
Three-cluster scenario
(a)
D. mentalis
1
1
0.89
1
1
northern Atlantic Forests
Central - southern Atlantic Forests
Andes - Tepuis Central America
SDTFs Southern Amazon
Outgroup
(b)
T. caerulescens
1
1
northern Atlantic Forests
Central - southern Atlantic Forests
central Andes
Outgroup
(c)
Two-cluster scenario
Three-cluster scenario
T. palliatus/ tenuepunctatus
*samples of T. tenuepunctatus
1
1
1
Atlantic Forests
Andes
Amazonian
Outgroup
(d)
Four-cluster scenario
Three-cluster scenario
T. ruficapillus/ torquatus
*T. torquatus
**T. ruficapillus
A 'taxonomic' scenario
1
1
1
1
1
1
northern Atlantic Forests
Central - southern Atlantic Forests
Central Andes
Bolivian montane dry forest - Southern Yungas
Outgroup
